# JUNB O‐GlcNAcylation‐Mediated Promoter Accessibility of Metabolic Genes Modulates Distinct Epithelial Lineage in Pulmonary Fibrosis

**DOI:** 10.1002/advs.202406751

**Published:** 2024-12-15

**Authors:** Marie‐Therese Bammert, Meshal Ansari, Leoni Haag, Zuhdi Ahmad, Victoria Schröder, Joseph Birch, Diana Santacruz, Werner Rust, Coralie Viollet, Benjamin Strobel, Alec Dick, Florian Gantner, Holger Schlüter, Fidel Ramirez, Muriel Lizé, Matthew J. Thomas, Huy Q. Le

**Affiliations:** ^1^ Lung Repair & Regeneration Department Boehringer Ingelheim Pharma GmbH & Co. KG 88400 Biberach Germany; ^2^ Faculty of Biology University of Konstanz 78457 Konstanz Germany; ^3^ Global Computational Biology and Digital Science Boehringer Ingelheim Pharma GmbH & Co. KG 88400 Biberach Germany; ^4^ Drug Discovery Sciences Boehringer Ingelheim Pharma GmbH & Co. KG 88400 Biberach Germany; ^5^ C.H. Boehringer Sohn AG and Co. KG 55218 Ingelheim Germany; ^6^ University of Bath Bath BA27JX UK

**Keywords:** aberrant epithelial remodeling, bronchiolization, JUNB, O‐GlcNAcylation, pulmonary fibrosis

## Abstract

Idiopathic pulmonary fibrosis (IPF) is a lethal disease with substantial unmet medical needs. While aberrant epithelial remodeling is a key factor in IPF progression, the molecular mechanisms behind this process remain elusive. Harnessing a 3D patient‐derived organoid model and multi‐omics approach, the first inventory of the connection between metabolic alteration, chromatin accessibility, and transcriptional regulation in IPF aberrant epithelial remodeling is provided. This remodeling is characterized by an increase in chromatin accessibility, particularly at JUNB motif‐enriched promoter regions proximal to transcription start sites of metabolic and pro‐fibrotic genes. Mechanistically, JUNB undergoes O‐linked β‐N‐acetylglucosamine modification (O‐GlcNAcylation), a critical step in modulating pro‐fibrotic responses to chronic injury. This modification is pivotal in fostering the emergence of aberrant epithelial basal cells in the alveolar niche, a proposed driver of IPF pathology. Specific deletion of O‐GlcNAcylation sites on JUNB attenuates the metaplastic differentiation of basal cells, thereby aiding in the restoration of the alveolar lineage. Together, the findings reveal a novel link between metabolic dysregulation and cell fate regulation at the chromatin level in fibrosis, mediated by the O‐GlcNAc‐JUNB axis, suggesting avenues for the development of new therapeutic strategies in IPF.

## Graphical abstract

Persistent injury and aberrant repair in idiopathic pulmonary fibrosis lead to the emergence of an aberrant basal cell phenotype. This cell fate is driven by metabolic dysregulation that increases O‐GlcNAc levels, specifically on JUNB. This alteration triggers the pro‐fibrotic response and aberrant repair. Perturbating O‐GlcNAcylation on JUNB reduces IPF hallmarks and promotes regenerative capacities.

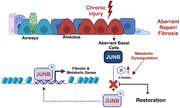

## 1 Introduction

The provision of cellular energy requirements is crucial in maintaining tissue homeostasis. This is achieved through cellular metabolism, which not only transforms nutrients into energy but also provides critical signals for other cellular activities, including proliferation, differentiation, immune response, and cytokine secretion.^[^ ^1^, ^2^, ^3^, ^4^ ^]^ These metabolic pathways are stringently regulated by a myriad of enzymes and signaling cascades, ensuring the fulfillment of the varied biological processes in accordance with their metabolic state. Any dysregulation within these processes can lead to injury and diseases.^[^ ^5^ ^]^ One prominent metabolic rheostat – O‐linked β‐N‐acetylglucosamine (O‐GlcNAc) – is a unique nutrient‐ and stress‐sensing glycosylation that functionally modifies a broad spectrum of proteins.^[^ ^6^ ^]^ O‐GlcNAc is transferred via the O‐GlcNAc transferase (OGT) to serine and threonine residues of target proteins and is removed by the O‐GlcNAcase (OGA), reflecting a unique dynamic property for post‐translational glycosylation. OGT utilizes uridine‐diphosphate N‐acetylglucosamine (UDP‐GlcNAc) as a precursor, which originates from several nutrient‐converting pathways, i.e., glycolysis, amino acid, or fatty acid metabolism. Regulatory proteins subject to O‐GlcNAcylation are involved in the spatiotemporal regulation of a broad spectrum of cellular processes, from transcriptional regulation to stress response, to maintain tissue homeostasis. Dysregulation in O‐GlcNAcylation is associated with certain pathologies including cancer, fibrosis, and ageing.^[^ ^7^, ^8^ ^]^

Idiopathic pulmonary fibrosis (IPF) is a rare, devastating disease, characterized by the excessive accumulation of extracellular matrix, leading to distortion of the alveoli and increased stiffness of the lung together with the loss of gas‐exchange surface.^[^ ^9^ ^]^ IPF patients have a poor prognosis, with an estimated survival time of two to three years post‐diagnosis. While fibroblasts were classically regarded as the major drivers of IPF progression, with most research and therapeutic strategies relying on targeting this cell population, an effective cure remains elusive. Importantly, accumulating evidence has shifted this perspective, suggesting IPF as predominantly an epithelial‐driven condition.^[^ ^10^, ^11^, ^12^, ^13^, ^14^ ^]^ Recurrent and unresolved epithelial injury leads to aberrant activation of basal cells, marked by KRT5+/KRT17+/COL1A1+, promoting the pro‐fibrotic cell fates and bronchiolization process in the diseased lung.^[^ ^15^, ^16^ ^]^ However, the underlying mechanisms shaping this disease phenotype and the fibrotic cell fate of lung epithelial progenitors/stem cells remain poorly understood, highlighting a critical gap in current IPF research.^[^ ^17^, ^18^ ^]^

Harnessing a patient‐derived 3D distal airway epithelial organoid model, we found that metabolic dysregulation, specifically through aberrant O‐GlcNAc, determines the pro‐fibrotic cell fate in IPF. This dysregulation shapes the hallmarks of IPF including expression of pro‐fibrotic markers, increased proliferative capacity, and presence of metaplastic aberrant basal cells by JUNB‐O‐GlcNAc. By inhibiting O‐GlcNAcylated JUNB, we successfully mitigated these important fibrotic characteristics. This discovery illuminates a novel path in which dysregulated metabolism translates into cell fate decisions. Furthermore, it suggests a new therapeutic approach for IPF by targeting aberrant O‐GlcNAc modifications on disease‐related factors as shown in ex vivo human precision‐cut lung slices, offering a promising avenue for the treatment of IPF and other age‐related diseases.

## 2 Results

### 2.1 Patient‐Derived Distal Airway Organoids Recapitulate IPF Phenotype

**Figure 1:**
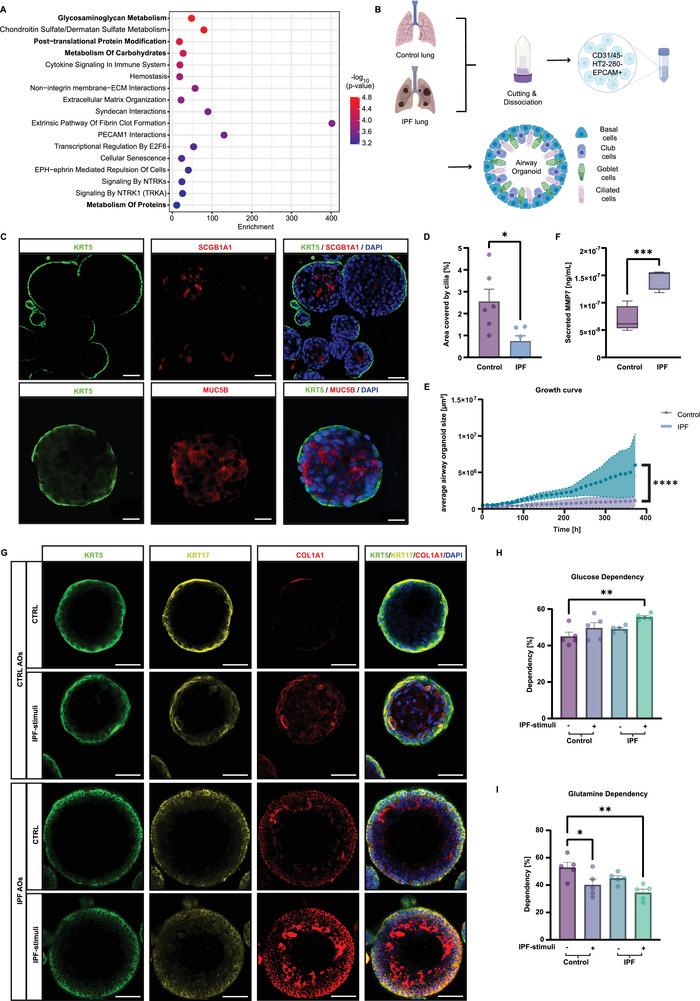
IPF‐derived distal airway organoids exhibit aberrant basal cell characteristics. A) Reactome analysis of gene signatures in aberrant basal cells shows a strong inclination toward glucose‐related metabolic pathways. B) Schematic representation of the cell isolation process from human distal lung tissue to generate patient‐derived AOs. C) Representative immunofluorescence staining of 15 days AOs shows a pseudostratified epithelium with basal cells (KRT5+, green) externally, and differentiated club cells (SCGB1A1+, red) and goblet cells (MUC5B+, red) internally. Nuclei are stained with DAPI (blue). Scale bars are 20 µm (first row) and 50 µm (second row). D) Quantification of ciliated cell area indicates a significant decrease in cilia in IPF AOs from n = 6 IPF and n = 6 control AO donors (mean + s.e.m, *p < 0.05, unpaired *t*‐test). E) Organoid size quantification over 15 days shows an increased proliferative capacity in IPF AOs (n = 4 IPF and n = 7 control AO donors, mean + s.e.m, ****p < 0.0001, unpaired *t*‐test). F) Profibrotic biomarker MMP7 secretion is increased in IPF AOs (n = 5 IPF and n = 5 control AO donors, mean + s.e.m., ***p < 0.001, unpaired *t*‐test). G) Representative immunofluorescence staining of AOs reveals aberrant basal cells (KRT5+(green)/KRT17+ (yellow)/COL1A1+ (red)). Nuclei are stained with DAPI (blue). Scale bars: 50 µm. IPF‐stimuli further enriches this population after 7 days. H, I) Seahorse XF Mito Fuel Flex Test kit showed increased glucose (H) and decreased glutamine (I) dependency of IPF and IPF‐stimulated AOs (n = 5 IPF and n  =  5 control donors, mean + s.e.m., *p < 0.05, **p < 0.01, ANOVA/ Tukey's).

To investigate the functional ramifications of aberrant epithelial activation in the pathogenesis of IPF, we revisited an integrated published single‐cell RNA sequencing (scRNA‐seq) data set with three independent sets of IPF patient lungs,^[^ ^19^ ^]^ with a focus on aberrant basal cells (**Figure** **1**A; Table S1, Supporting Information). Alongside the involvement of canonical fibrotic pathways, we observed a significant enrichment of metabolic pathways, predominantly glucose‐related, and of post‐translational protein modifications within this subpopulation. To further investigate the role of metabolic dysregulation in IPF aberrant basal cells, we utilized a 3D organoid system, derived from lung tissue samples obtained from IPF patients and healthy, control resections from tumor explants. We first isolated EPCAM+ distal airway epithelial cells through tissue dissection and multiple sorting steps to obtain a pure epithelial cell fraction (Figure 1B). The harvested cells were cultured in Cultrex basement membrane extract to promote the formation and growth of distal airway organoids (AOs).^[^ ^20^ ^]^ Under these culture conditions, AOs displayed a uniform, spherical morphology with a lumen and exhibits a pseudostratified epithelial layer encompassing functional basal, club, goblet, and ciliated cells (Figure 1C; Figure S1A–C, Supporting Information). Markers for basal (KRT5+), goblet (MUC5B+), and club cells (SCGB1A1+) were detected within 14 days of development (Figure 1C; Figure S1A, Supporting Information), while cilia (FOXJ1+) became visible after 21 days (Figure S1A, B, Supporting Information). Intriguingly, a reduction in ciliated area was observed in IPF AOs compared to control (Figure 1D).

Through time‐lapse imaging, we noted that IPF AOs exhibited rapid initial growth (Figure 1E; Figure S1D, E, Supporting Information), reflecting the progressive nature of fibrotic disease.^[^ ^10^, ^21^ ^]^ Additionally, nanoindentation measurements on cells derived from IPF AOs showed increased stiffness of the epithelium (Figure S1F, Supporting Information), falling within the range of an IPF lung.^[^ ^22^, ^23^ ^]^ This was accompanied by elevated levels of secreted MMP7, an IPF clinical biomarker^[^ ^24^ ^]^ (Figure 1F).

Importantly, aberrant basal cells were detected in IPF AOs through immunofluorescence staining for KRT5, KRT17, and COL1A1, in contrast to control AOs (Figure 1G). This phenotype was intensified upon exposure to pro‐fibrotic stimuli.^[^ ^25^ ^]^ Moreover, we identified a metabolic transition favoring heightened glucose dependency and diminished glutamine dependency (Figure 1H, I; Figure S1G, Supporting Information), as expected in fibrotic disease.^[^ ^26^, ^27^, ^28^ ^]^

In summary, our findings demonstrate the successful generation of functional patient‐derived organoids that closely reflect key hallmarks of IPF, including aberrant basal cells, metabolic dysregulation, disease‐associated biomarkers, and elevated matrix production, providing a valuable platform for studying disease mechanisms.

### 2.2 IPF Progression is Associated with Increased O‐GlcNAcylation in Aberrant Basal Cells

**Figure 2:**
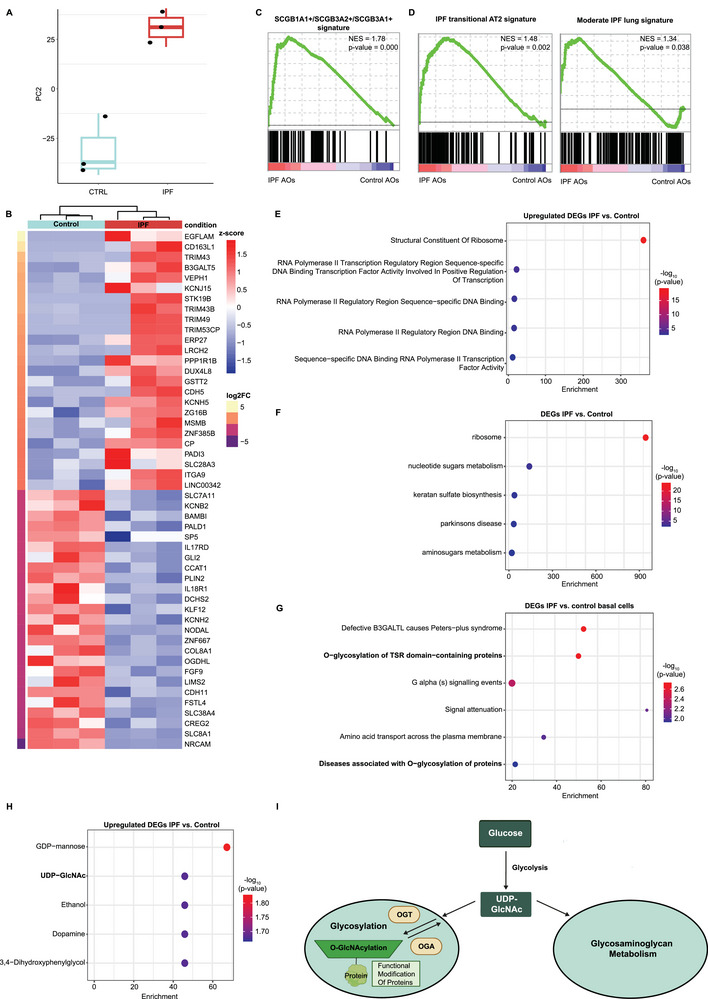
O‐GlcNAc plays an important role in aberrant basal cells in IPF. A) Principal component analysis (PCA) for PC‐2 of RNA‐seq data with the top 5000 variable transcripts shows distinct clustering of IPF and control AOs (boxplot, n = 3 control and n = 3 healthy AO donors, p‐value < 0.05). B) Hierarchical clustering of the top 50 differentially expressed genes (DEGs) from RNA‐seq between IPF and control AOs (p‐value < 0.05). C) GSEA using cell type signature database (c8) shows enrichment of a secretory signature (SCGB1A1+/SCGB3A2+/SCGB3A1+) in IPF AOs (p‐value < 0.05). D) GSEA shows enrichment of an IPF transitional AT2 signature and moderate IPF lung stage signature in IPF AOs using IPF database (p‐value < 0.05). E) Gene ontology (GO) molecular function analysis shows enrichment of increased transcriptional machinery activity in IPF AOs (p‐value < 0.05, log2FC > 0). F) KEGG analysis shows enrichment of metabolic pathways, specifically glycosylation‐related, among all DEGs in IPF AOs (p‐value < 0.05). G) REACTOME analysis reveals enrichment of disease‐associated O‐glycosylation in IPF basal cells (scRNA‐seq data, p‐value < 0.05, log2FC > 0, n = 2 control and n = 2 IPF AOs). H) Metabolomics workbench metabolites analysis shows enrichment of UDP‐GlcNAc metabolite in IPF AOs (p‐value < 0.05, log2FC > 0). I) Schematic overview of UDP‐GlcNAc and its relation to the glycosaminoglycan metabolism and glycosylation comprising O‐GlcNAcylation. The precursor UDP‐GlcNAc is reversibly added to proteins via OGT and OGA to induce O‐GlcNAcylation on targeted proteins.

To delve deeper into the molecular mechanisms governing aberrant basal cell fate, we analyzed transcriptional profiles of IPF and control AOs using RNA sequencing. Upon evaluation of the transcriptomic profile, we observed distinct clustering of the respective conditions and identified many IPF‐associated differentially expressed genes (DEGs; p‐value < 0.05) in IPF AOs (**Figure** **2**A–B; Table S2A, Supporting Information). This divergence was clearly enhanced upon exposure to pro‐fibrotic stimuli (Figure S2A–C, Supporting Information). Using an unbiased phenotypical signatures analysis, we observed an overrepresented signature of secretory cells characterized by SCGB1A1+/SCGB3A2+/SCGB3A1+^[^ ^29^ ^]^ in IPF AOs (Figure 2C; Table S2B, Supporting Information). Notably, these cells have been previously described to be prevalent as a transitional state within severely fibrotic regions of the lung.^[^ ^30^, ^31^, ^32^ ^]^ This prompts us to investigate the resemblance of the IPF AOs to human diseases by incorporating clinical data from various stages of IPF.^[^ ^33^ ^]^ Gene set enrichment analysis (GSEA) unveiled a significant enrichment of gene signatures associated with IPF transitional alveolar type 2 (AT2)^[^ ^19^ ^]^ and lung profiles from patients with moderate IPF^[^ ^33^ ^]^ (Figure 2D; Table S2C, Supporting Information). Together, this data corroborates the relevance of the IPF AO model to IPF, particularly in terms of the classic histological description of the bronchiolization process.

Next, we proceeded to explore the differences in gene expression between the IPF and control AOs. Among the most significantly enriched gene ontology (GO) terms in the upregulated DEGs of IPF AOs were those related to RNA polymerase II transcription machinery and transcription factors activities (Figure 2E; Table S2D, Supporting Information). This indicates a potential transcriptional reconfiguration in IPF. Consistent with the scRNA‐seq findings from IPF lungs (Figure 1A), pathway analysis further revealed the engagement of various metabolic pathways in IPF AOs, particularly those associated with glycosylation and glycosaminoglycan metabolism (Figure 2F; Figure S2D, E and Table S2E, Supporting Information). These results were further corroborated by data from an additional 5 control and 5 IPF donors (Figure S2F, Supporting Information). Importantly, focusing on aberrant basal cells specifically highlighted over‐represented changes in O‐glycosylation (Figure 2G; Table S2F–G and Figure S2G, Supporting Information). This is intriguing as these metabolic processes are known to mediate transcriptional reprogramming through several routes, including the post‐translational modifications (PTMs) of transcription factors.^[^ ^34^ ^]^ This prompted us to delineate the specific PTMs induced by these metabolic pathways. Through metabolic regulation analysis that clusters gene sets by their common metabolites, we identified an enrichment of UDP‐GlcNAc in IPF AOs (Figure 2H; Table S2H, Supporting Information). This metabolite is a pivotal precursor for O‐GlcNAcylation; a unique and reversible type of O‐glycosylation PTM, and a building block in glycosaminoglycan metabolism processes^[^ ^35^, ^36^ ^]^ (Figure 2I). Collectively, these findings propose that dysregulation of O‐GlcNAc modifications would modulate the aberrant cell behavior, thereby exacerbate the progression of IPF specifically.

### 2.3 Increased O‐GlcNAc Levels Drive IPF Progression

**Figure 3:**
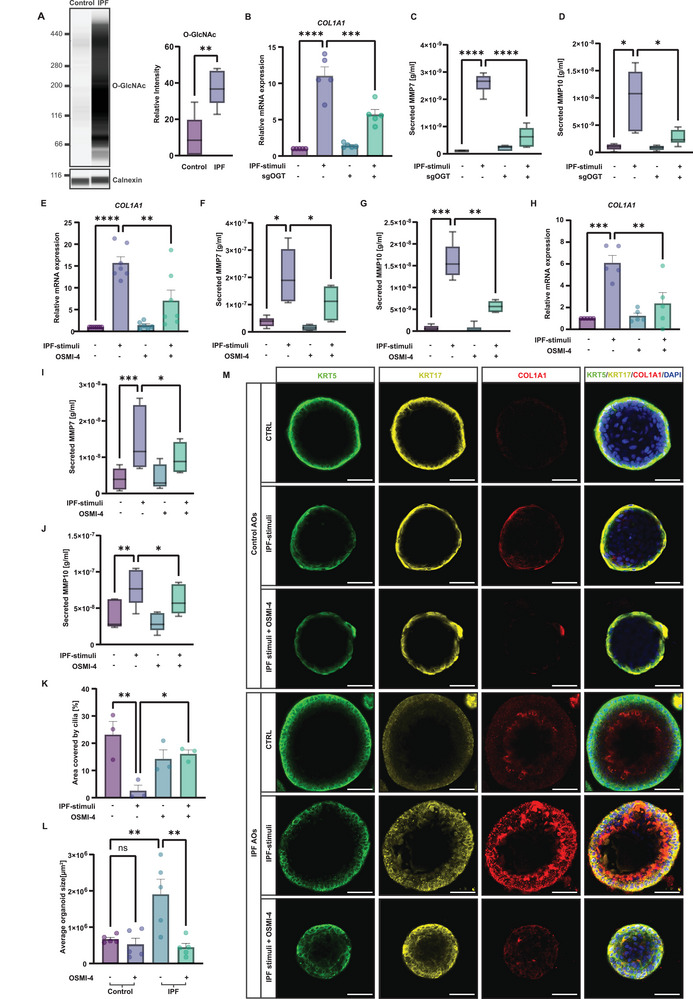
OGT inhibition reduces fibrotic characteristics and aberrant basal cell fate. A) Representative western blot analysis and quantification (right panel) of total O‐GlcNAc levels in control (n = 6) and IPF (n = 5) lung lysates revealed significant increase of O‐GlcNAc in IPF lungs (violin plot, **p < 0.01, unpaired *t*‐test). B) RT‐PCR analysis of *COL1A1* in OGT‐deleted airway epithelial cells treated with IPF‐stimuli shows that OGT is required for profibrotic gene expression (n = 5, mean + s.e.m, *p < 0.05, ****p < 0.0001, ANOVA/Tukey's). C, D) ELISA analysis represents a decrease in pro‐fibrotic protein secretion of MMP7 (C) and MMP10 (D) in OGT‐deleted airway epithelial cells stimulated with IPF‐stimuli. (n = 8, boxplot, *p < 0.05, ****p < 0.0001, ANOVA/ Tukey's). E) RT‐PCR analysis of *COL1A1* in IPF AOs treated with IPF‐stimuli and OSMI‐4 for 7 days shows decrease of gene expression upon OGT inhibition (n = 7, mean + s.e.m, **p < 0.01, ****p < 0.0001, ANOVA/Tukey's). F, G) ELISA analysis shows a decline in MMP7 (F) and MMP10 (G) secretion in an OGT‐dependent manner upon stimulation with IPF‐stimuli (n = 6, boxplot, *p < 0.05, **p < 0.01, ***p < 0.001 ANOVA/Tukey's). H) RT‐PCR analysis shows that OGT inhibition attenuates chronically injured epithelial‐fibroblast coculture induced *COL1A1* expression levels (n = 5, mean + s.e.m, **p < 0.01, ***p < 0.001, ANOVA/Tukey's). I, J) ELISA analysis reveals that OGT inhibition in chronically injured epithelial‐fibroblast coculture ameliorates MMP7 (I) and MMP10 (J) secretion (n = 5, boxplot, *p < 0.05, **p < 0.01, ***p < 0.001, ANOVA/ Holm‐Šídák's). K) Quantification of ciliated cell area upon OSMI‐4 treatment and chronic injury in epithelial‐mesenchymal coculture increased area covered by ciliated cells (n = 3, mean + s.e.m, *p < 0.05, **p < 0.01, ANOVA/Tukey's). L) Quantification of average organoid size shows decreased organoid growth upon inhibition of OGT after 10 days (n = 5, mean + s.e.m, **p < 0.01, ANOVA/Tukey's). M) Representative immunofluorescence staining of IPF and control AOs treated with IPF‐stimuli and OSMI‐4 for 7 days shows decrease in aberrant basal cell signature upon OGT inhibition (scale bar 50 µm).

To test this hypothesis, we measured global O‐GlcNAc levels in patient‐derived lung lysates and detected a remarkable increase in IPF lungs (**Figure** **3**A). To access the regulatory role of increased O‐GlcNAc levels in IPF, we deleted OGT (Figure S3A, Supporting Information). As expected, OGT depletion attenuated the expression of pro‐fibrotic genes and proteins in IPF‐stimulated airway epithelial cells (Figure 3B–D; Figure S3B, Supporting Information). Similar effects were observed within IPF‐induced fibroblasts (Figure S3C–E, Supporting Information), indicating a conserved regulatory role of aberrant O‐GlcNAc levels in IPF cell fate decisions.

Subsequent to our hypothesis, we sought to explore the therapeutic potential of modulating OGT activity by employing the small molecule inhibitor OSMI‐4.^[^ ^37^ ^]^ First, we tested the effect of this inhibitor on O‐GlcNAcylation levels in 2D and 3D distal airway epithelial cultures and determined half‐maximal effective concentrations (EC50) of 3 µM and 10 µM, respectively (Figure S3F–I, Supporting Information). Intriguingly, inhibition of O‐GlcNAc modifications by OSMI‐4 culminated in a marked diminution of pro‐fibrotic marker expression and secretion in both, IPF AOs (Figure 3E–G; Figure S3J, Supporting Information) and epithelial‐fibroblasts co‐cultures (Figure 3H‐J; Figure S3K–L, Supporting Information). Furthermore, OSMI‐4 treatment not only effectively mitigated and restored the loss of ciliated cells in IPF (Figure 3K), but also significantly attenuated the proliferative capacity of IPF AOs (Figure 3L). This observation aligns with previous findings which show anti‐proliferative effects of inhibited O‐GlcNAc levels in a variety of cancer cells.^[^ ^38^, ^39^ ^]^

Remarkably, treatment with OGT inhibition in the presence of an IPF stimuli in IPF AOs led to a substantial reduction in aberrant basal cells, achieving levels comparable to those observed in control organoids (Figure 3M), highlighting its potential to address both epithelial cell loss and abnormal proliferation and activation associated with IPF.

Collectively, these experiments suggest the central role of O‐GlcNAcylation in governing the aberrant cell fate decisions in IPF. The pharmacological inhibition of this post‐translational modification by OSMI‐4 not only mitigates fibrotic features but also appears to restore the epithelial integrity by reducing metaplastic aberrant basal cells and rescuing the loss of ciliated cell populations, thereby providing a compelling therapeutic avenue for ameliorating the pathological manifestations of IPF.

### 2.4 IPF is Associated with Increased Chromatin Accessibility at Promoter Regions of Metabolic Genes

**Figure 4:**
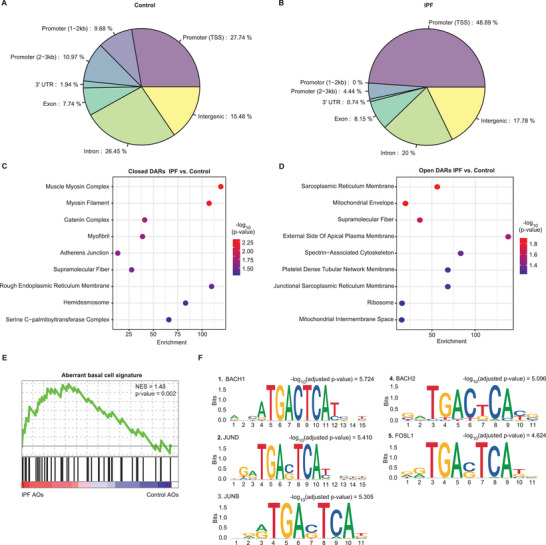
JUNB is enriched at promoter regions of open chromatin in IPF. A, B) Genomic annotations of IPF basal cells in AOs for significantly differentially closed (A) and open (B) accessible regions (DARs, p*‐value* < 0.05) shows enrichment of open chromatin in IPF at promotor and transcription start site (TSS) regions. Genomic regions are defined as introns, exons, intergenic regions, 3′ UTR, promotor TSS (defined as peak summits within 1 kb upstream and downstream of the TSS), promotor (1‐2 kb), and promotor (2‐3 kb). C,D) Gene ontology (GO) enrichment analysis of closed (C) and open (D) DARs using annotated genes (*q*‐value < 0.05) in IPF basal cells. Open regions were associated with signal transduction and metabolic pathways, whilst closed regions were related to mechanical stress and barrier function. E) GSEA reveals enrichment of annotated genes based on DARs (*q‐value* < 0.05) for aberrant basal cell signature in IPF basal cells. F) Top 5 transcription factor (TF) motif enrichments in IPF basal cells.

To delineate the fundamental alterations within the chromatin landscape that contribute to the O‐GlcNAc‐induced aberrant cell fates in IPF, we profiled the chromatin accessibility profiles using Assay for Transposase‐Accessible Chromatin sequencing (ATAC‐seq) in IPF and control AOs on a single‐cell level focusing on the basal cell fraction (Figure S4A–C, Supporting Information). Following the read alignment process and subsequent peak calling, we assessed the distribution of peaks with respect to established genomic features (**Figure** **4**A, B; Table S3A, Supporting Information). Despite the similarity in peak distribution across different conditions, the proximal promoter and transcription start site (within 1 kb) regions exhibited significantly greater accessibility in IPF AOs compared to control (48.89% and 27.74%, respectively).

GO enrichment analysis of significantly differentially accessible regions (DARs) revealed that IPF AOs were associated with decreased chromatin accessibility at promoter regions of genes responsible for mechanical stretch and barrier function (Figure 4C; Table S3B, Supporting Information), suggesting that these key regulatory genes, which are vital for maintaining lung tissue integrity and response to mechanical forces, become less active, contributing to the impaired lung function seen in IPF. Conversely, regions associated with metabolism and signal transduction gained accessibility (Figure 4D; Table S3C, Supporting Information). Additionally, these altered accessible regions showed a significant correlation with the IPF aberrant basal cell signature (Figure 4E; Table S3D, Supporting Information), indicating a reprogramming of gene regulatory network and cellular priorities that may offer insights into disease etiology.

Next, to gain insight into the regulatory network of open chromatin accessibility in IPF aberrant basal cells, we conducted transcription factor (TF) motif enrichment analysis and found significantly overrepresented motifs specific to the activator protein 1 (AP1) complex in the IPF condition (Figure 4F). The presence of these TFs, which have been reported to play essential roles in regulating gene expression in response to stress,^[^ ^40^, ^41^, ^42^ ^]^ implicates their contribution to the pathophysiology of IPF. Similar findings were also observed in a larger cohort of IPF donors within using a bulk ATAC‐seq analysis (Figure S4D–K and Table S3E, Supporting Information).

Collectively, our findings revealed that IPF is characterized by increased chromatin accessibility and gene expression, particularly at promoter regions with the motif of AP1 transcription factor family members that control pro‐fibrotic cell fate decisions.

### 2.5 O‐GlcNAcylation Determines Pro‐Fibrotic Cell Fate Decisions Through JUNB

**Figure 5:**
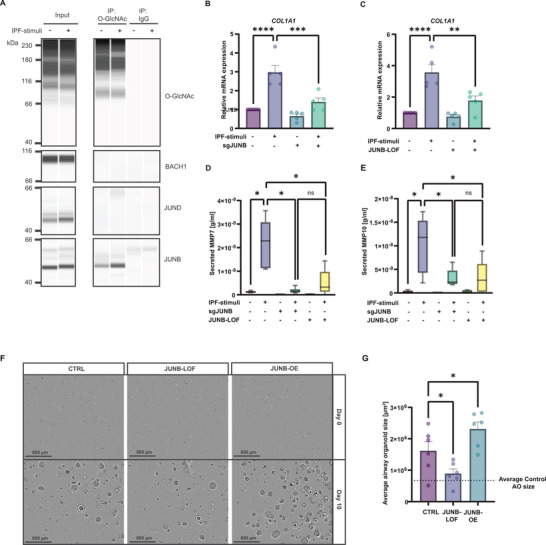
O‐GlcNAcylation of JUNB induces pro‐fibrotic cell fate decisions. A) Representative western blot analysis of the O‐GlcNAc immunoprecipitated fraction shows increased levels of O‐GlcNAc mark on JUNB in injured airway epithelial cells, but not on BACH1 and JUND (n = 5 donors). IgG antibody was employed as negative control. Input and immunoprecipated fraction have been sliced together. B, C) RT‐PCR analysis shows diminished expression of *COL1A1* in JUNB‐deleted (B) and JUNB‐LOF (loss‐of‐function) (C) transfected and fibrotic‐induced airway epithelial cells (n  =  5, mean + s.e.m, *p < 0.05, **p < 0.01, ****p < 0.0001, ANOVA/Tukey's). Plasmid was generated by site‐specific mutations in the O‐GlcNAc sites of JUNB. D, E) Transduction of JUNB‐LOF plasmid or deletion of JUNB in fibrotic‐induced airway epithelial cells leads to a decrease in the secretion of MMP7 (D) and MMP10 (E) (n = 6, boxplot, *p < 0.05, ANOVA/ Holm‐Šídák's). F, G) Representative pictures (F) and quantification (G) of average organoids size of n = 6 IPF AOs was reduced after JUNB‐LOF transduction in AOs after 10 days, whereas overexpression of JUNB led to a significant increase in growth (mean + s.e.m, *p < 0.05, ANOVA/Tukey's).

Previous studies discovered that TFs could carry O‐GlcNAc modifications, which influence their transcriptional activity and stability,^[^ ^34^ ^]^ therefore we hypothesize that O‐GlcNAcylation of AP1 family members may serve as a stress sensor to regulate pro‐fibrotic response in the lung. To evaluate this hypothesis, we examined the presence of O‐GlcNAc modification on the top three enriched TFs identified in our analysis: BACH1, JUND, and JUNB. Immunoprecipitation (IP) showed an increase in O‐GlcNAc PTM levels on JUNB upon pro‐fibrotic stimulation in airway epithelial cells, but not in BACH1 and JUND (**Figure** **5**A). We further assessed whether the expression of these TF motifs exhibited differential expression in IPF and found that JUNB was significantly upregulated in IPF aberrant basal cells^[^ ^43^ ^]^ (Figure S5A, Supporting Information). The increase of O‐GlcNAc modification on JUNB was also detected in fibroblasts (Figure S5B, Supporting Information) further emphasizing the critical role of aberrant O‐GlcNAcylation in modifying JUNB and its potential involvement in the pro‐fibrotic signaling.

To validate the specific role of O‐GlcNAcylated JUNB in driving the fibrotic cell fate, we substituted the potential O‐GlcNAc sites on JUNB^[^ ^44^ ^]^ from serine to alanine (S‐to‐A) and threonine to valine (T‐to‐V) via site‐specific mutations,^[^ ^45^ ^]^ and expressed this loss‐of‐function (LOF) of O‐GlcNAc sites on JUNB in airway epithelial cells (plasmid sequence in Table S4, Supporting Information). Upon exposure to IPF‐stimuli, this modification led to a significant attenuation in the expression of pro‐fibrotic genes and secreted proteins, producing effects comparable to depleting JUNB expression (Figure 5B–E; Figure S5C–D, Supporting Information). Furthermore, expression of JUNB‐LOF in human primary fibroblasts treated with IPF‐stimuli exerts a reduction in the pro‐fibrotic response, suggesting the effect of O‐GlcNAcylated JUNB extends beyond epithelial cells in driving pro‐fibrotic responses (Figure S5E–I, Supporting Information).

We next assessed the role of O‐GlcNAcylated JUNB using IPF AOs. We evaluated the average organoid growth over a 10‐day period post adeno‐associated viral (AAV) transduction with a JUNB‐LOF construct and an overexpression of JUNB (JUNB‐OE). The JUNB‐LOF resulted in a significant reduction in organoid size after 10 days, reaching levels similar to those seen in control AOs (Figure 5F–G). In contrast, JUNB overexpression stimulated organoid growth. Together, these data underline the involvement of O‐GlcNAcylated JUNB in IPF pathology, particularly its pivotal role in steering the pro‐fibrotic response in the lung.

### 2.6 Increased O‐GlcNAcylation Levels Compromise Alveolar Differentiation Ex Vivo

Finally, we investigated the impact of this machinery rheostat within the context of more complex native lung tissue using an ex vivo model of rat precision cut lung slices (rPCLS) and human PCLS (hPCLS). This system retains the intricate cellular diversity and spatial architecture inherent to the lung, providing a robust platform for pharmacological investigations in near native conditions, as emphasized in recent studies.^[^ ^46^, ^47^ ^]^ We induced fibrosis through the administration of a species‐specific IPF‐relevant cocktail (IPF‐RC).^[^ ^48^ ^]^

**Figure 6:**
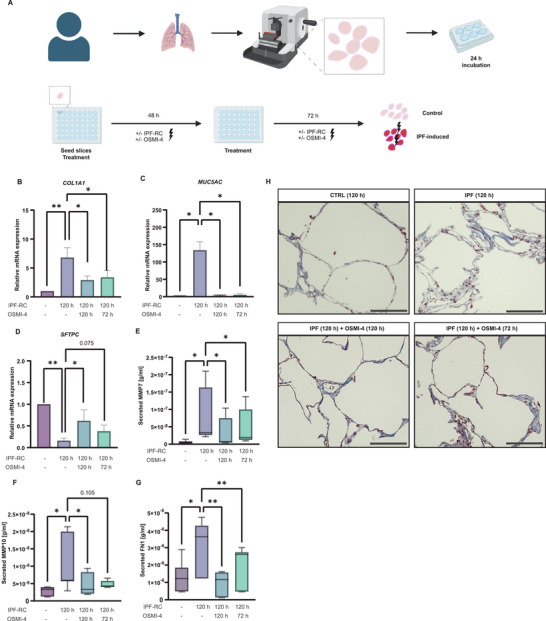
O‐GlcNAcylation mediates epithelial cell fates in an ex vivo model. A) Schematic overview of generation and treatment of hPCLS. Human lungs were filled with agarose and lung tissue dissected. Slices were cut with a microtome to a thickness of 200–350 µm. Afterwards, slices were incubated for 24 h prior to seeding and treatment with ± IPF‐RC and ± OSMI‐4 for 48 h followed by additional treatment for another 72 h (total treatment time: 120 h). B, C) RT‐PCR analysis of *COL1A1* (B) and MUC5AC (C) in hPCLS treated with OSMI‐4 for 120 h showed reduction of pro‐fibrotic marker upon IPF‐RC stimulation (n = 5 human lungs, mean + s.e.m, *p < 0.05, **p < 0.01, ANOVA/Tukey's). D) RT‐PCR analysis of *SFTPC* in hPCLS treated with OSMI‐4 for 120 h reflects increase in alveolar regeneration upon IPF‐RC stimulation (n = 5 human lungs, mean + s.e.m, *p < 0.05, **p < 0.01, ANOVA/Friedman). E, F) ELISA analysis reveals decreased expression of pro‐fibrotic markers MMP7 (E) and MMP10 (F) upon stimulation with OSMI‐4 (n = 7 human lungs, mean + s.e.m, *q < 0.05, ANOVA/Friedman with FDR correction). G) ELISA analysis showed decreased expression of pro‐fibrotic marker FN1 upon stimultation with OSMI‐4 (n = 7 human lungs, mean + s.e.m, *p < 0.05, **p < 0.01, ANOVA/Holm‐Šídák's). H) Representative Masson Trichrome stainings of hPCLS slices after respective treatment for 120 h shows reduction of lung architecture distortion upon OSMI‐4 treatment for 120 h and 72 h (scale bar 150 µm).

Following a 24‐h incubation period post slicing, PCLS of both species were subjected to the respective treatment with IPF‐RC and OSMI‐4 for a total of 120 h (**Figure** **6**A; Figure S6A, Supporting Information). We first verified that the elevated O‐GlcNAc levels in fibrosis were preserved in rat lung tissues (Figure S6B, Supporting Information). Importantly, the induction of a fibrotic phenotype was successfully achieved as evidenced by an upregulation in pro‐fibrotic and mucus‐associated markers and a concurrent decrease in the AT2 cell marker surfactant protein c (*SFTPC)* in both rat and human PCLS (Figure 6B–G; Figure S6C–F, Supporting Information).

Simultaneous inhibition of O‐GlcNAc for 120 h mitigated the pro‐fibrotic and mucous response and augmented *SFTPC* expression (Figure 6B–G; Figure S6C–F, Supporting Information), suggesting the prevention of IPF hallmarks by OSMI‐4. Remarkably, this effect was also replicated in a therapeutic setting, where OSMI‐4 was added only in the last 48 h of the experiment. Specific targeting of O‐GlcNAc on JUNB by transduction with the JUNB‐LOF showed comparable effects and led to a decrease in pro‐fibrotic markers and strongly increased alveolar gene expression (Figure S6G–J, Supporting Information). Histological analysis revealed that stimulation with IPF‐RC distorted the normal lung structure (Figure 6L; Figure S6K, Supporting Information). These structural changes were ameliorated with OSMI‐4 treatment for 120 h and 72 h, respectively.

These findings indicate that reducing O‐GlcNAc levels in pulmonary fibrosis not only diminishes pro‐fibrotic responses, but also enables alveolar repair and regeneration in injured lung tissue, highlighting the therapeutic promise of targeting O‐GlcNAc in IPF.

## 3 Discussion

**Figure 7:**
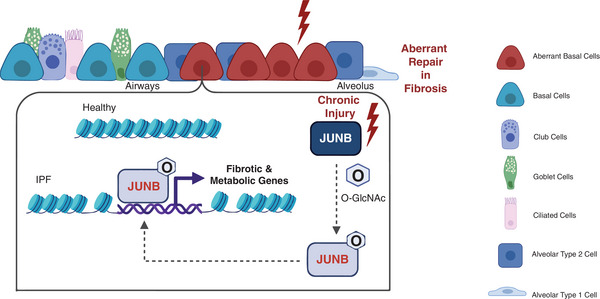
Schematic model describes the expansion of aberrant basal cells driven by the O‐GlcNAC‐JUNB axis. Chronic injury within the lung epithelium fosters an aberrant basal cell phenotype, which in turn elevates global O‐GlcNAc levels, and O‐GlcNAcylation of JUNB. The O‐GlcNAc‐JUNB drives the fibrotic cell fate by accessing open promotor regions of metabolic and pro‐fibrotic genes to promote aberrant repair and fibrosis.

In the disease state, cells adopt a unique metabolic state, distinct from their healthy counterparts, to sustain their survival in a hostile pathologic microenvironment.^[^ ^49^ ^]^ This adaptation involves the use of various metabolic pathways across different cell types, indicating a complex regulatory mechanism driving disease progression. In this study, we demonstrate how a metabolic shift impacts chromatin accessibility and transcriptional reprogramming of aberrant basal cells in driving the bronchiolization process in IPF, as summarized in **Figure** **7**.

While human organoids have commonly been employed to study genetic diseases, our data highlights the use of a 3D in vitro organotypic disease model for IPF, derived from patient tissue with non‐genetic upstream pathological stimuli. Remarkably, this model successfully recapitulates important hallmarks of IPF, including increased tissue stiffness, the emergence of aberrant basal cells, and metabolic dysregulation.^[^ ^50^, ^51^ ^]^ Interestingly, tissue mechanics are known to influence the chromatin landscape and thus stem cell function in ageing^[^ ^52^ ^]^ and age‐related diseases.^[^ ^53^ ^]^ To the best of our knowledge, chromatin landscape profiling for epithelial bronchiolization in IPF has not been analyzed. Our findings suggest that tissue stiffening could trigger a series of molecular events, including a metabolic shift crucial for stem cell functions such as activation, proliferation, and migration. This is regulated through the O‐GlcNAc‐JUNB axis, which enhances chromatin accessibility and transcriptional reprogramming, ultimately leading to the aberrant behavior and metaplastic proliferation of KRT5+ basal cells in alveolar regions.

Elevated O‐GlcNAc levels are a hallmark of a variety of diseases,^[^ ^35^ ^]^ including fibrosis^[^ ^54^ ^]^ and cancer,^[^ ^55^ ^]^ yet their precise targets are elusive. Here, we observe increased O‐GlcNAc levels in the bronchiolization process in IPF. Together with previous research on fibroblasts,^[^ ^54^ ^]^ our findings indicate that O‐GlcNAcylation may play a crucial role in the development and progression of fibrosis throughout the lung. Importantly, JUNB, a part of the activator protein 1 transcription factor family, has been implicated in a myriad of biological processes^[^ ^56^, ^57^, ^58^, ^59^ ^]^ and disease pathogenesis, particularly in cancer.^[^ ^41^, ^60^ ^]^ However, its role is controversial, and has been classified both as a tumor suppressor^[^ ^61^ ^]^ and an oncogene.^[^ ^41^, ^42^ ^]^ Our findings introduce a concept that O‐GlcNAcylation may fundamentally alter cellular activity of JUNB, shedding new light on the connection between metabolism and stress response. Depending on its O‐GlcNAc status, it may switch JUNB activity across diverse environmental conditions, as it was previously observed for MORC2.^[^ ^62^ ^]^ Intriguingly, inhibiting the O‐GlcNAc modification on JUNB attenuates the metaplastic differentiation of KRT5+ aberrant basal cells and induces regenerative features, such as the decrease of aberrant basal cells in IPF AOs. Moreover, by utilizing a highly recognized ex vivo model for pharmacological investigations under in vivo‐like conditions for human lungs, we were able to expand our findings and detect the re‐emergence of alveolar cell markers, accompanied by a reduction in fibrotic biomarkers, and an overall improvement in the histological structure of the lung tissue. The use of a human PCLS model further demonstrates the translation of our findings to clinical context, providing additional evidence for the relevance of altered O‐GlcNAcylation in IPF. The therapeutic benefits seen from using OSMI‐4 to block global O‐GlcNAc levels could extend beyond the effects on JUNB; the role of O‐GlcNAc‐modifications in the context of IPF is still largely unexplored and warrants further investigations. This highlights the importance of future studies aimed at mapping the O‐GlcNAcome implicated in IPF. Additionally, the development of more targeted inhibitors for O‐GlcNAcylation of JUNB could pave the way for novel treatment strategies.

Collectively, our study sheds light on how metabolic changes might convert into established signaling pathways through O‐GlcNAc, which serves as a modulator of JUNB, promoting fibrotic cell fate decisions and aberrant basal cell invasion. This insight offers a new avenue for selectively targeting this specific modification, as opposed to broadly inhibiting TF activity, presenting a viable therapeutic approach to arrest fibrotic progression and stimulate lung regeneration.

### 3.1 Limitations of the Study

This study has revealed the mechanism of aberrant O‐GlcNAcylation on JUNB in IPF using patient‐derived organoids and human PCLS. While PCLS serve as a valuable tool for clinically relevant research, verifying this mechanism in animal model is essential for understanding systemic effects. AP1 family members are reported as proto‐oncogenes and require a specific in vivo laboratory under gene safety level 2, to which we do not have access and so could not evaluate this in an in vivo model. This requires further investigations.

## 4 Experimental Section

### Ethical Approval

Patient lung tissue was obtained from the German Centre for Lung Research (DZL/BREATH) Hannover in Germany with full local ethical approval by DZL's ethics committee (10 194_B0_K_2022) and patient consent. Further airway organoid donors were purchased at HUB Organoids as ready‐to‐use cell stocks with ethical approval and patient consent.

All human tissue samples are coded / de‐identified. Studies were performed in accordance with the local legislation and institutional requirements.

### Human Lung Tissue Processing and AO Cultivation

To isolate human lung cells, tissue samples are collected from the distal lung and cut into 2 mm slices. Subsequently, the tissues are enzymatically treated and placed into the gentleMACS dissociator to induce cell detachment. From the obtained cell suspension dead cells are removed and erythrocytes are depleted. Eventually, the remaining cells are magnetically sorted out with MACS. First, endothelial cells (CD31/45+ cells) are collected. From the remaining cell suspension, alveolar cells (HT2‐280+ cells) are sorted out, yielding the last fraction from which the distal airway epithelial cells are obtained (EpCAM+ cells). Next, the EpCAM+ cells are seeded in Cultrex RGF BME type 2 with airway organoid media to support organoid formation and expansion (based on Sachs et al.^[^ ^20^ ^]^). Once organoids have fully formed, they are cultivated depending on the experimental set‐up.

To further induce fibrosis in AOs, an IPF‐relevant cytokine cocktail (IPF‐RC)^[^ ^25^ ^]^ was used 5x concentrated for 7 days. Composition of 5x IPF‐RC is listed in Table S5 (Supporting Information). To block OGT in AOs a commercially available inhibitor (OSMI‐4, MedChemExpress #HY‐114361^[^ ^63^ ^]^) was used in 10 µM concentration for 7 days.

### 2D Cultivation of Primary Cells

Healthy human lung airway epithelial cells (SAECs, Lonza, #CC‐2547, or patient‐derived distal airway epithelial cells) were used in passages 2–3 and expanded on type I collagen (Corning, #354 236) pre‐coated cell culture flasks in PneumaCult‐Ex Plus Medium (STEMCELL Technologies, #0 5040). For air‐liquid‐interface differentiation (ALI), airway epithelial cells were seeded on collagen‐coated transwells, grown until confluency, and airlifted by changing medium to PneumaCult‐ALI Medium (STEMCELL Technologies, #0 5001) and exposing cells apically to air. Differentiated cells were observed after 10 days.

Healthy human lung fibroblasts (LF) (Lonza, #CC‐2512) were used in passages 4–8 and expanded in flasks in Fibroblast Basal Medium (Lonza, #CC‐3131) with FGM2‐Fibroblast Growth Medium BulletKit (Lonza, CC‐3132).

For pro‐fibrotic induction 3 ng mL^−1^ TGF‐β1 and for OGT inhibition 3 µM OSMI‐4 was used for 72 h.

### Epithelial‐Mesenchymal Chronic Injury Coculture

SAECs were differentiated in air liquid interface (ALI) on transwells for 10 days. Afterwards, ALI SAECs were treated for 14 days with 1 ng mL^−1^ TGF‐β1 or vehicle to induce chronic injury and cultivated in coculture with human lung fibroblasts ± 1 ng mL^−1^ TGF‐β1 in the basolateral chamber for additional 3 days.

### Histology

AOs were seeded and cultivated as previously described. After 25 days, AOs were dissolved away from Cultrex RGM Type 2 (R&D systems, #3533‐010‐02) and fixed in 4% paraformaldehyde (PFA) for 20 min at RT. PCLS were treated as described and afterwards fixed for 30 min in 4% PFA at RT. Afterwards cells and PCLS were washed and resuspended or covered with HistoGel (Richard‐Allan Scientific, #HG‐4000‐012) and transferred to a paraffin cassette and incubated for 30 min at 4 °C. Next, the cassette was embedded in paraffin and cut into 3 µm slices by using microtome. For subsequent Hematoxylin‐Eosin, Masson Trichrome, or Periodic‐Acid‐Schiff staining, the respective AO or PCLS sections were deparaffinized and further stained according to standard protocols.

### Immunofluorescence Staining

For immunofluorescence staining, control and IPF AOs were seeded on µ‐slide 8‐well chamber slides (IBIDI, #80 807), and cultivated or treated as previously described. AOs were fixed with 4% PFA for 30 min and permeabilized with 0.2% TritonX‐100 in PBS, followed by blocking in 1% BSA in PBS. Samples were incubated overnight at 4 °C with the respective primary antibody, washed with PBS, and incubated with secondary antibodies and DAPI (Thermo Fisher, #62 248). After final washing, samples were covered with PBS and imaged with either the LSM710 or LSM900 (Zeiss) confocal microscope using 20x, 40x, or 63x immersion objectives. Images were evaluated using ZEN software3.7 (Zeiss). Intensity was equally increased using Adobe Photoshop (Adobe).

Primary antibodies used: mouse anti‐Mucin‐5AC CF488 conjugated (Invitrogen, #MA1‐38223; LOT:) rabbit anti‐Keratin‐5 Alexa Fluor 647 conjugated (Abcam, #ab193895; 1:100); rat anti‐SCGB1A1 (Novus Biological, #MAB4218‐SP; 1:50); rabbit anti‐KRT5 (Cell Signaling, #25 807; 1:500); guinea pig anti‐Keratin‐17 (Progen, #GP‐CK17, 1:300); mouse anti‐Collagen 1a1 (Sigma Aldrich, #SAB4200678, 1:100).

Secondary antibodies used (all from Invitrogen): anti‐rat immunoglobulin‐G (IgG) Alexa Fluor (AF) 568 (#A‐11077; 1:500); anti‐rabbit IgG AF488 (#A‐11034, 1:500); anti‐guinea pig IgG AF568 (#A11075; 1:500); anti‐mouse IgG AF647 (#A21235, 1:500).

### Nanoindentation

The stiffness of airway epithelial cells derived from AOs and seeded on transwells was measured via interferometry‐based optical fiber‐top nanoindentation by Pavone (Optics11 Life, Amsterdam, Netherlands). Cantilevers with stiffnesses of 0.029 N m^−1^ and 0.017 N m^−1^ and an area of 32.5 µm^2^ was applied. Prior to the measurement, a matrix scan was performed where nanoindentation of 30 points per well was performed.

### RNA Extraction and Quantitative RT‐PCR

Total RNA was extracted using the RNeasy Plus Micro kit (Qiagen, #74 034) and concentration was measured by NanoDrop. Reverse transcription into cDNA was performed using the SuperScript VILO cDNA Synthesis Kit (Invitrogen, #11 754 050). Gene expression analysis was carried out by using TaqMan assays and TaqMan Advanced Master Mix (Applied Biosystems, #4 444 557). Samples were measured in triplicates on the Quantstudio 6 Real Time PCR machine. Changes in gene expression were determined using the comparative cycle threshold method with housekeeper genes RPS26, GAPDH, and B2M.

The following human (h) and rat (r) TaqMan assays (Thermo Fisher) were used: *hKRT5* (Hs00361185_m1); *hSCGB1A1* (Hs00171092_m1); *hMUC5B* (Hs00861595_m1); *hFOXJ1* (Hs00230964_m1); *hKRT17* (Hs00356958_m1); *hCOL1A1* (Hs00164004_m1); *hOGT* (Hs00269228_m1); *hFN1* (Hs01549976_m1); *hCOL5A1* (Hs00609133_m1); *hJUNB* (Hs00357891_s1); *hSFTPC* (Hs00951326_g1); *hMUC5AC* (Hs01365616_m1); *hRPS26* (Hs00955682_g1); *hGAPDH* (Hs02786624_g1); *hB2* *M* (Hs00187842_m1); *rCol1a1* (Rn07363068_m1), *rFN1* (Rn00569575_m1); *rMuc5ac* (Rn01451252_m1); *rSftpc* (Rn00569225_m1); rGapdh (Rn01775763_g1)

### IncuCyte Organoid Growth Assay

To determine average organoid size over time, singularized organoids were seeded in a concentration of 1000 cells/µl and cultivated according to organoid cultivation protocol. For AAV6.2 induced JUNB‐LOF, JUNB‐OE, and GFP‐control expression (Table S4, Supporting Information), AOs were seeded and subsequently transduced with AAV vectors for 24 h at a MOI of 10^5^. Images were taken every 12 h for 10–15 days by using IncuCyte Live Cell Analysis system with a 4x Plan‐Apochromat objective in organoid assay mode. Analysis was conducted using IncuCyte Live Cell Analysis software.

### ELISA

Supernatants were centrifuged and diluted (MMP7/MMP10 1:10‐100; FN1: 1:200‐500). Measurement of human MMP7 (Biotechne, #DY907), human MMP10 (Biotechne, #DY910), and human FN1 (Invitrogen, #BMS2028) were performed according to manufacturer's instructions. Relative fluorescence intensity (excitation 490 nm, emission 520 nm) was measured via SpectraMax M5 and SoftMax Pro software (Molecular Devices). Total protein concentration was determined by the standard curve.

### Cilia Beating Measurement

Active beating cilia were calculated from image stacks of 2D+time as previously described.^[^ ^64^ ^]^ In brief, four different regions evenly distributed over the transwell were imaged using 32x objective of an Axiovert 25 microscope (Zeiss) and an acA 1300‐200 µm black and white USB‐3.0 high speed camera (Basler). Movement of cilia was recorded by taking 100 frames per second for a total of 6 seconds per transwell area (total of 512 images with a size of 512*512 pixels). Applications for image and analysis were developed by using HALCON 13.0.2 (MVTec Software) machine vision software toolbox and to visualize the image stack Analyze (AnalyzeDirect) was applied. Area covered by active cilia was calculated by quantification of pixels which were detectably moving within the chosen area.

### Seahorse Mito Fuel Flex Test

Seahorse Mito Fuel Flux Assays were performed using XFe96 Bioanalyzer (Agilent). Airway epithelial cells were seeded on collagen‐coated XFe96 cell culture plates and cultivated for 48 h. Afterwards, 5x IPF‐RC was used for 72 h and the assay performed according to manufacturer's instructions (Agilent, #103260‐100). Calculations of Capacity [%] and Dependency [%] were performed using Wave Software V2.6.3.5 based on OCR measurements (Agilent).

### Western Blot

To determine protein expression levels, simple western automated western blot systems PeggySue, SallySue, or Jess (Biotechne) were used according to manufacturer's instructions (Protein simple, Biotechne, #SM‐S001, #SM‐S002). Exposure was set to “High Dynamic Range”. Quantification was performed by normalization to calnexin or using Simple Western total protein quantification. To obtain proteins, tissue was lysed using T‐Per tissue extraction buffer or cells using RIPA buffer with phosphatase/protease inhibitor, respectively. Antibodies used: mouse anti‐OGlcNAc RL2 (Novus Biologicals, #NB300‐534, 1:500), mouse anti‐JUNB (Thermo Fisher, #SAB1406056, 1:50), mouse anti‐JUND (Biotechne, #MAB5526, 1:50), mouse anti‐BACH1 (Thermo Fisher, #66762‐1‐IG, 1:50) rabbit anti‐calnexin (Cell Signaling, #2679, 1:700).

### Immunoprecipitation

Cells were harvested in IP lysis buffer with phosphatase‐/protease‐inhibitor followed by short incubation and lysate clearance at 18 000 g. Protein concentration was determined using Pierce BCA assay (Thermo Fisher, # 23 225). Pre‐coupled antibody beads (Cell signaling, Custom Conjugation Service; rabbit anti‐O‐GlcNAc IgG (#82 332) and rabbit IgG isotype control (#3900)) were washed twice and taken up in IP lysis buffer. Next, they were mixed with 500 µg protein per reaction and incubated at 4 °C overnight. After repetitive washing with IP lysis buffer, proteins were eluted using Pierce IgG elution buffer (Thermo Fisher, #21 004) and neutralized by adding 1 M Tris buffer (pH 9.0, 1:100 in sample).

### RNA Extraction and RNA‐Seq

After taking out the respective number of cells for ATAC‐seq, cells were pelleted and lysed in RLT Plus buffer (QIAGEN, #1 053 393). Total RNA was extracted and purified on the MagMAX instrument (Thermo Fisher) using the MagMAX 96 Total RNA Isolation Kit (Thermo Fisher #AM1830) following manufacturer's instructions. Total RNA was quantitatively and qualitatively assessed using High Sensitivity dsDNA Quanti‐iT Assay Kit (ThermoFisher) on a Synergy HTX (BioTek) and High Sensitivity Total RNA Analysis DNF‐472 on a 96‐channel Fragment Analyzer (Agilent), respectively. All samples had RIN values >9. Total RNA samples were normalized on the MicroLab STAR automated liquid platform (Hamilton). A total RNA input of 250 ng was used for library construction with the NEBNext Ultra II Directional RNA Library Prep Kit for Illumina (#E7760), together with the NEBNext Poly(A) mRNA Magnetic Isolation Module (#E7490) and NEBNext Multiplex Oligos for Illumina (#E7600) (New England Biolabs). RNA‐seq libraries were processed on a Biomek i7 Hybrid instrument (Beckman Coulter) using 13 PCR cycles, following the manufacturer's protocol, except for the use of Ampure XP beads (Beckman Coulter) for double‐stranded cDNA purification and PCR clean‐up. Final sequencing libraries were quantified by the High Sensitivity dsDNA Quanti‐iT Assay Kit (ThermoFisher) on a Synergy HTX (BioTek). Libraries were also assessed for size distribution and adapter dimer presence (<0.5%) by the High Sensitivity NGS Fragment 1–6000 bp kit on a 96‐channel Fragment Analyzer (Agilent). All sequencing libraries were then normalized on the MicroLab STAR (Hamilton), pooled, and sequenced on an Illumina NovaSeq 6000 using an S2 Reagent Kit v1.5 (Run parameters: 101 bp, Rd2: 8 bp, Rd3: 8 bp, Rd4:101 bp) with an average sequencing depth of ≈30 million pass‐filter reads per sample.

### Analysis of Bulk RNA‐Seq

Differential expression analysis was carried out with DESeq2 (v1.42.0).^[^ ^65^ ^]^ Statistical tests were performed via the Wald test and p‐values were corrected with the Benjamini‐Hochberg method. Genes with a p‐value below 0.05 were considered differentially expressed (Table S2A, Supporting Information). Differential expressed genes were further analyzed using EnrichR^[^ ^66^, ^67^ ^]^ and GSEA.^[^ ^68^ ^]^

### Bulk ATAC Sample Preparation and Sequencing

For matched bulk‐ATAC and RNA‐seq three IPF‐ and control‐derived airway organoid donors were used, respectively, and singularized. ATAC‐seq library prep was performed as previously described^[^ ^69^ ^]^ with minor adaptions. In brief, 70 000 cells of singularized AOs were lysed in lysis buffer (10 mM Tris pH 7.5, 10 mM NaCl, 3 mM MgCl2, 0.1% NP‐40) for 5 min and centrifuged. Pelleted cells were transposed using Tagment DNA TDE1 Enzyme and Buffer Kit (Illumina, # 20 034 197) for 60 min at 37 °C with shaking. Afterwards, samples were purified using clean‐up buffer (0.5 M NaCl, 400 mM EDTA, 1% SDS, 2 mg mL^−1^ Proteinase K) and normal phase 2x AMPureXP (Beckman Coulter, #A63881) bead clean‐up, followed by PCR amplification. Finally, amplified samples were purified using DNA Clean & Concentrator Kit (Zymo, #ZYM‐D4029) and double‐sided clean‐up with AMPure beads. Libraries were quantitatively and qualitatively assessed using the 1x dsDNA kit on the Qubit 4 Fluorometer (ThermoFisher) and the High Sensitivity NGS Fragment 1–6000 bp kit on a 48‐channel Fragment Analyzer (Agilent), respectively. ATAC‐seq libraries were normalized on the MicroLab STAR (Hamilton), pooled, and spiked in with PhiX Control v3 (Illumina). Libraries were sequenced on an Illumina NovaSeq 6000 using an S2 Reagent Kit v1.5 (#20 028 315; Read parameters: Rd1:101 bp, Rd2: 8 bp, Rd3: 8 bp, Rd4:101 bp) with an average sequencing depth of ≈100 million reads passed filter per sample.

### Analysis of bulkATAC‐Seq

After sequencing we used the nf‐core ataq‐seq pipeline (v2.1.2)^[^ ^70^, ^71^ ^]^ to perform alignment and downstream analysis. Briefly, pre‐alignment quality control and adapter trimming of the FASTQ files was performed with the default parameters. The reads were then aligned to the human reference genome (GRCh38.86) and alignments from multiple libraries per sample were merged. The standard settings of the pipeline were kept to remove low‐quality reads, including PCR duplicates and reads mapping to mitochondrial DNA. Following peak calling, the consensus peak set across all samples was generated. To assess quality metrics, we employed functions from the deepTools package^[^ ^72^ ^]^ and explored the enrichment of read coverage (plotFingerprint) and visualized gene scores across genome regions (computeMatrix and plotProfile,).

Next, we extracted the peak count matrix and excluded peaks that were present in less than 5 samples. DESeq2 (v1.42.0)^[^ ^65^ ^]^ was used to identify differentially accessible peaks. The model was fitted to the count matrix using the DESeqDataSetFromMatrix function. Following statistical tests (Wald test) to compare read counts between the conditions of interest. Peaks with a q‐value (Benjamini‐Hochberg corrected) below 0.05 were considered differentially accessible (Table S3E, Supporting Information). Differentially accessible regions were annotated to their genomic regions using ChIPseeker (v1.40.0)^[^ ^73^ ^]^ and further analyzed using EnrichR^[^ ^66^, ^67^ ^]^ and GSEA.^[^ ^68^ ^]^

Motif enrichment analysis was conducted on genomic regions that exhibited increased accessibility following IPF or IPF‐treatment. The tool findMotifs.pl of the HOMER suite^[^ ^74^ ^]^ generated tables listing enriched motifs with statistical significance scores and their associated transcription factors. As a baseline for motif enrichment, the background sequences were randomly selected from the genome. To identify specific instances of the JUNB binding motif, we utilized the tool scanMotifGenomeWide.pl to search for matching positions across the genome.

### Nuclei Isolation and Simultaneous scATAC and snRNA‐Seq

For simultaneous scATAC and snRNA‐seq, AOs were singularized as previously described. Cells were resuspended in ice‐cold buffer (1x PBS + 0.04% BSA) and filtered through a 40 µm cell filter (Flowmi BelArt). Cell concentration, viability, and aggregate were determined with the NucleoCounter NC‐3000 (Chemometec) using Via‐1 cassette and on NucleoView NC‐3000 software (Chemometec, v.2.1). A total of 850 000 cells per sample, with an average cell viability of 92.2 ± 1.7% and cell aggregate <5%, were used for nuclei isolation using 10x Genomics Demonstrated Protocol for Nuclei Isolation for Single Cell Multiome ATAC + GEX Sequencing (CG000365‐RevC). Nuclei concentration was determined with the NucleoCounter NC‐3000 using A2 slides and a double staining of Acridine Orange and DAPI (Solution 13, Chemometec). 4 single‐nuclei suspensions were employed to generate scATAC and snRNA‐seq libraries that were prepared with the Chromium Next GEM Single Cell Multiome kit ATAC + Gene Expression according to the manufacturer's instructions (CG000338 Rev E, 10x Genomics). Samples were loaded into a NextGEM Chip J (10x genomics) with a targeted nuclei recovery of 8000 nuclei. Capture and GEM generation was conducted with the Chromium controller followed by reverse transcription, Post‐GEM Cleanup, and a Pre‐amplification of 8 cycles. After Pre‐Amplification SPRI Cleanup, each sample was divided and used as input for separate scATAC and snRNA‐seq library preparations. For scATAC‐seq, 35 µL from the purified Pre‐Amplification eluate was used for cDNA amplification with 12 cycles and purified with SPRI beads. Qualitative and quantitative measurement of cDNA was conducted with the High Sensitivity NGS Fragment 1–6000 bp kit on a 48‐channel Fragment Analyzer (Agilent) and 1x dsDNA kit on the Qubit 4 Fluorometer (ThermoFisher), respectively. For snRNA‐seq, a total of 250 ng of cDNA per sample was employed for library preparation with an additional 1x SPRISelect bead clean‐up (Beckman Coulter) to ensure full removal of primer and adaptor dimers prior to the final elution. snRNA‐seq and scATAC‐seq libraries were amplified with 12 and 7 PCR cycles, respectively, and quantified with the High Sensitivity dsDNA Quanti‐iT Assay Kit (ThermoFisher) using a Synergy HTX microplate reader (BioTek) and qualitatively assessed with the High Sensitivity NGS Fragment 1–6000 bp Kit on a 96‐channel Fragment Analyzer (Agilent). Final libraries were normalized, pooled, spiked in with 5% PhiX Control v3 (Illumina), and sequenced on an Illumina Novaseq 6000 at a depth of ≈50 000 reads per cell with dual index, paired end reads (snRNA‐seq Read parameters: Rd1: 28 bp, Rd2: 10 bp, Rd3: 10 bp Rd4: 91 bp, scATAC‐seq Read parameters: Rd1: 50 bp, Rd2: 8 bp, Rd3: 24 bp Rd4: 49 bp).

### Computational Processing of the Multiome Data

De‐multiplexed scRNA‐seq and scATAC‐seq FASTQ files were run in the Cell Ranger ARC pipeline (10X Genomics, v2.0.2) to produce barcoded count matrices and fragment files. GRCh38.86 was used as reference genome for alignment of the reads. The quality control and analysis were performed on each modality separately.

### Analysis of scATAC‐Seq

The downstream analysis of the scATAC‐seq data was performed with the R package ArchR (v1.0.2).^[^ ^75^ ^]^ The basic input for ArchR are Arrow files, a tool‐specific data structure that stores data associated with an individual sample (i.e., metadata, accessible fragments, and data matrices). The tab‐separated fragment files from the cellranger ARC run were the basis to generate such Arrow files for each of the four input samples. Common quality control metrics such as the number of unique nuclear fragments, the fragment size distribution, and transcription starting site enrichment were inspected. Potential doublet cells were filtered out with filterDoublets and the parameter filterRatio set to 1.5.

For dimensionality reduction we used Latent Semantic Indexing (LSI) as suggested in the common workflow due to the high level of sparsity inherent to ATAC‐seq data. LSI was calculated via addIterativeLSI, using the “TileMatrix” as input with the following parameters: iterations = 2, resolution = 0.2, sampleCells = 10 000, varFeatures = 25 000, dimsToUse = 1:30. As we expected sample‐specific effects, batch correction was performed with Harmony^[^ ^76^ ^]^ based on the LSI space. The 2D embedding was generated on the Harmony‐corrected space via addUMAP with the parameters nNeighbors = 40, minDist = 1, metric = “cosine”. We calculated cluster specific markers with getMarkerFeatures and default parameters and assigned cell population identity based on common cell type marker of lung airway epithelial cells (MUC5B, SCGB1A1, KRT5, KRT15). The cell type annotation was used as basis for peak calling with addReproduciblePeakSet which is based on the tool macs2.^[^ ^77^ ^]^

We subset to the basal cell population to perform differential accessibility analysis comparing IPF‐derived cells to donor cells (getMarkerFeatures with useMatrix = “PeakMatrix”, groupBy = “health_status”, testMethod = “Wilcoxon”, bias = “TSSEnrichment” and “log10(nFrags)”) (Table S3A, Supporting Information).

Differentially accessible regions of basal cells were annotated to their genomic regions and further analyzed as described for bulkATAC‐seq (Table S3B‐D, Supporting Information). Finally, we aimed to infer which transcription factors may mediate the creation of these accessible chromatin sites. Thus, motif enrichment analysis was performed with peakAnnoEnrichment by providing differentially accessible sites in IPF patients as input (FDR < 0.1 and log2 foldchange ≥ 0,5).

### Analysis of scRNA‐Seq

The scRNA‐seq counterpart was processed and analyzed with the Scanpy package (v1.10.2).^[^ ^78^ ^]^ Quality control was performed after assessing established quality metrics in the data set. For barcode filtering, we excluded barcodes with less than either 500 transcripts or 200 detected genes. Further, cells with mitochondria‐encoded counts (above 25%) were excluded. Genes that were present in less than 5 cells were discarded as well. Doublet filtering was performed sample‐wise by the scrublet extension within Scanpy^[^ ^79^ ^]^ and cells with a predicted doublet score of less than 0.3 were retained. This resulted in 30 733 cells after filtering. The expression matrices were normalized and log‐transformed using Scanpy functions pp.normalize_total() and pp.log1p(), respectively. The list of top 1000 genes was calculated with pp.highly_variable_genes() by providing the sample IDs as batch parameter. Known cell cycle as well as mitochondrial genes were excluded from this list, resulting in a total of 850 variable genes. This list formed the input for dimensionality reduction. We employed scVI (v1.1.5)^[^ ^80^ ^]^ to calculate a batch‐corrected latent space and provided the first 10 dimensions as input to nearest‐neighborhood graph construction via tl.neighbors(n_neighbors = 20) and UMAP calculation via tl.umap(). After clustering the cells with the Leiden method at resolution 1, cell type populations were annotated based on manual curation of known airway epithelial marker. Differential gene expression in the basal cells was performed at pseudo bulk level with the package pydeseq2 (v0.4.10)^[^ ^81^ ^]^ (Table S2F, Supporting Information).

### Transfections (CRISPR‐Cas9/Plasmids)

2D cultured airway epithelial cells or mesenchymal cells were transfected via electroporation with Amaxa Nucleofector 2b device (Lonza) and the respective kit, (Lonza, #VPI‐1005 or) at program T‐020 according to manufacturer's instructions to introduce ribonuclear‐protein‐complex (RNP) complex, plasmids, or control, respectively. The following TrueGuide Synthetic gRNAs (Thermo Fisher, #A35533) were used: OGT (CRISPR887744_SGM); JUNB (CRISPR924803_SGM, CRISPR924804_SGM ratio 1:1 mixed); TrueGuide sgRNA Negative Control (#A35526).

### AAV6.2 Production

Self‐complementary AAV6.2 vectors were produced and purified as previously described.^[^ ^82^ ^]^ Briefly, high‐density HEK‐293 h cells (AcCELLerate) were seeded into 8‐layer CELLdiscs and triple‐transfected with pHelper, pRep2/Cap6.2 and JUNB‐encoding plasmids (Table S4, Supporting Information) using the Calcium phosphate procedure. Following culture for 72 h, cells were lysed by three freeze/thaw cycles under high salt conditions. Following salt‐active nuclease treatment and PEG precipitation, AAVs were purified by iodixanol step gradient ultracentrifugation, followed by Amicon‐15‐based buffer exchange/ultrafiltration and sterile filtration. Genomic titers were determined by dPCR (Qiagen), using ITR‐specific primers.

AOs were infected after seeding with AAV constructs for 24 h at a MOI of 10^5^.

### Precision Cut Lung Slices (PCLS)

Male rats (Wistar Han) were purchased from (Charles River). Rats were housed in individually ventilated cages at 22–25 °C, a humidity of 46–65%, and 12‐h day/night cycle. Animals received water and food ad libitum. Ethical approval for this study was obtained from the regional govern‐mental animal care and use office (Regierungspräsidium Tübingen, Germany, TVV 18‐030‐O).

Healthy rats were euthanized using 60 mg kg^−1^ Pentobarbital‐sodium (Narcoren). Diaphragm was punctured and lungs were infiltrated with ≈20 mL of LM agarose using a cannula with syringe. Human tissue was processed accordingly. Immediately, agarose‐filled lungs were cooled with ice for 15 min. Afterwards, ice was removed, the lobes were separated and cut with a microtome (Krumdieck) to a thickness of 200–350 µm on medium blade and movement speed. Once slicing was completed, slices were transferred to 6‐well plate inserts with 10 mL medium (1x DMEM/F12 + L‐glutamine + 15 mM HEPES + 1x Antibiotica‐Antimycotica + 0.1% fetal bovine serum) and cultivated at 37 °C, 5% CO2. The next day, slices were distributed in a 48 well plate with 300 µL medium including respective treatment. After 48 h, medium was exchanged, and slices cultivated another 72 h for a total cultivation time of 144 h.

For the transduction of JUNB‐LOF in PCLS, slices were infected with 5×10^10^ VG mL^−1^ per slice and co‐stimulated with IPF‐RC for 48 h, followed by another infection and treatment for 72 h (total cultivation: 120 h).

### RNA and Protein Extraction of PCLS

Slices were collected in Precellys Lysis Matrix D tubes and snap‐frozen in liquid nitrogen. Afterwards, protein was lysed in T‐Per (+phosphatase/protease inhibitor) buffer, RNA was lysed using RLT Plus buffer and samples were homogenized for 25 s at 6500 rpm on a Precellys device (Bertin Technologies).

For protein, samples were incubated on ice for 15 min and again homogenized with the previous settings. Next, samples were cleared at 18 000 g for 15 min, followed by simple western analysis.

For RNA, homogenates were incubated for 5 min at RT and transferred to PhaseLock tubes with phenol/chloroform/isoamyl alcohol (25:24:1). After shaking, samples were centrifuged at 16 000 g for 5 min and chloroform/isoamyl alcohol (24:1) was added followed again by shaking. Next, samples were incubated for 3 min at RT and centrifuged again for 10 min. The extracted RNA was then purified and processed for RT‐PCR as previously described.

### Statistical Analysis

GraphPad prism (version 10.1.2) was used to perform statistical analysis. The following tests were used to determine significance: Unpaired t‐test, paired t‐test with Wilcoxon test, one‐way ANOVA with Holm‐Šídák's, Šídák's, Tukey's, FDR or Dunn's test, non‐linear/linear regression. Gaussian distribution was determined for all tests, which required normal distribution, with the Kolmogorov‐Smirnov test (α = 0.05). At least three biological replicates were performed for all experiments.

## Conflict of Interest

All authors were employed by Boehringer Ingelheim Pharma GmbH & Co KG or by C.H. Boehringer Sohn AG and Co KG. This study was funded by Boehringer Ingelheim Pharma GmbH & Co KG. HQL is currently employed by Bayer AG.

## Author Contributions

M.B. designed, performed, and supervised most of the experiments and analyzed data. M.B., D.S., W.R., and A.D. performed sequencing experiments. M.A. and F.R. analyzed sequencing data. Z.A., L.H., and V.S. performed experiments. M.B. and J.B. performed ex vivo experiments. M.J.T., M.L., H.S., F.G., B.S., and C.V. provided conceptual advice. H.Q.L. conceived, conceptualized, supervised the study, and designed experiments. M.B. and H.Q.L. wrote the paper. All authors commented on and edited the manuscript.

## Supporting information



Supporting Information

Supplemental Table 1

Supplemental Table 2

Supplemental Table 3

Supplemental Table 4

Supplemental Table 5

## Data Availability

ATAC‐ and RNA‐seq data generated within this study was deposited in the Gene Expression Omnibus and will be made accessible upon acceptance of the manuscript. All other data is available on request.

## References

[1] J. Zhu , C. B. Thompson , Nat. Rev. Mol. Cell Biol. 2019, 20, 436. 30976106 10.1038/s41580-019-0123-5 PMC6592760

[2] L. A. J. O'Neill , R. J. Kishton , J. Rathmell , Nat. Rev. Immunol. 2016, 16, 553. 27396447 10.1038/nri.2016.70 PMC5001910

[3] B. T. Jackson , L. W. S Finley , Cell Stem Cell 2024, 31, 161. 38306993 10.1016/j.stem.2024.01.003 PMC10842269

[4] A. Marrocco , L. A. Ortiz , Front. Immunol. 2022, 13, 936167 . 36341426 10.3389/fimmu.2022.936167 PMC9633986

[5] R. J. DeBerardinis , C. B. Thompson , Cell 2012, 148, 1132 . 22424225 10.1016/j.cell.2012.02.032 PMC3337773

[6] Y. Xiaoyong , Q. Kevin , Nat. Rev. Mol. Cell Bio. 2017, 18, 452 .28488703

[7] F. Charlie , A. H. John , Nat. Chem. Biol. 2022, 18, 8.34934185

[8] K. Zhang , R. Yin , X. O.‐G. N. A. c. A. B. S. L Yang , Front. Endocrinol. 2014, 5, 221. 10.3389/fendo.2014.00221 PMC4269194 25566193

[9] S. L. Barratt , A. Creamer , C. Hayton , N. Chaudhuri , J. Clin. Med. 2018, 7, 201. 30082599 10.3390/jcm7080201 PMC6111543

[10] A. Camelo , R. Dunmore , M. A. Sleeman , D. L. Clarke , Front. Pharmacol. 2014, 4, 173. 24454287 10.3389/fphar.2013.00173 PMC3887273

[11] P. Confalonieri , M. C. Volpe , J. Jacob , S. Maiocchi , F. Salton , B. Ruaro , M. Confalonieri , L. Braga , Cells 2022, 11. 10.3390/cells11132095 PMC9266271 35805179

[12] I. T. Stancil , J. E. Michalski , D. Davis‐Hall , H. W. Chu , J.‐A.h Park , C. M. Magin , I. V. Yang , B. J. Smith , E. Dobrinskikh , D. A. Schwartz , Nat. Commun. 2021, 12, 4566 . 34315881 10.1038/s41467-021-24853-8 PMC8316442

[13] I. Shakeel , M. Afzal , A. Islam , S. S. Sohal , M.d. I. Hassan , Idiopathic pulmonary fibrosis: Pathophysiology, cellular signaling, diagnostic and therapeutic approaches 2023, 20, 100167.

[14] T. Parimon , C. Yao , B. R. Stripp , P. W. Noble , P. Chen , Int. J. Mol. Sci. 2020, 21, 2269. 32218238 10.3390/ijms21072269 PMC7177323

[15] B. Jaeger , et al., Airway basal cells show a dedifferentiated KRT17highPhenotype and promote fibrosis in idiopathic pulmonary fibrosis 2022, 13, 5637. 10.1038/s41467-022-33193-0 PMC9513076 36163190

[16] R. J. Hewitt , F. Puttur , D. C. A. Gaboriau , F. Fercoq , M. Fresquet , W. J. Traves , L. L. Yates , S. A. Walker , P. L. Molyneaux , S. V. Kemp , A. G. Nicholson , A. Rice , E. Roberts , R. Lennon , L. M. Carlin , A. J. Byrne , T. M. Maher , C. M. Lloyd , Nat. Commun. 2023, 14, 6039 . 37758700 10.1038/s41467-023-41621-y PMC10533905

[17] P. Saha , P. Talwar , Idiopathic pulmonary fibrosis (IPF): disease pathophysiology, targets, and potential therapeutic interventions 2024, 479, 2181. 10.1007/s11010-023-04845-6 37707699

[18] H. Guo , J. Sun , S. Zhang , Y. Nie , S. Zhou , Y. Zeng , Front. Pharmacol. 2023, 14, 1205948. 37608885 10.3389/fphar.2023.1205948 PMC10440605

[19] C. H. Mayr , L. M. Simon , G. Leuschner , M. Ansari , J. Schniering , P. E. Geyer , I. Angelidis , M. Strunz , P. Singh , N. Kneidinger , F. Reichenberger , E. Silbernagel , S. Böhm , H. Adler , M. Lindner , B. Maurer , A. Hilgendorff , A. Prasse , J. Behr , M. Mann , O. Eickelberg , F. J. Theis , H. B. Schiller , EMBO Mol. Med. 2021, 13, 12871. 10.15252/emmm.202012871 PMC8033531 33650774

[20] N. Sachs , A. Papaspyropoulos , D. D. Zomer‐van Ommen , I. Heo , L. Böttinger , D. Klay , F. Weeber , G. Huelsz‐Prince , N. Iakobachvili , G. D. Amatngalim , J. de Ligt , A. van Hoeck , N. Proost , M. C. Viveen , A. Lyubimova , L. Teeven , S. Derakhshan , J. Korving , H. Begthel , J. F. Dekkers , K. Kumawat , E. Ramos , M. F.m van Oosterhout , G. J. Offerhaus , D. J. Wiener , E. P. Olimpio , K. K. Dijkstra , E. F. Smit , M. van der Linden , S. Jaksani , et al., EMBO J. 2019, 38, 100300. 10.15252/embj.2018100300 PMC6376275 30643021

[21] A. Chakraborty , M. Mastalerz , M. Ansari , H. B. Schiller , C. A. Staab‐Weijnitz , Cells 2022, 11. 10.3390/cells11061050 PMC8947093 35326501

[22] F. Liu , J. D. Mih , B. S. Shea , A. T. Kho , A. S. Sharif , A. M. Tager , D. J. Tschumperlin , J. Cell Biol. 2010, 190, 693. 20733059 10.1083/jcb.201004082 PMC2928007

[23] T. Guo , C. He , A. Venado , Y. Zhou , Compr Physiol 2022, 12, 3523. 35766837 10.1002/cphy.c210032 PMC10088466

[24] Y. Bauer , E. S. White , S. de Bernard , P. Cornelisse , I. Leconte , A. Morganti , S. Roux , O. Nayler , ERJ Open Res 2017, 3, 00074. 28435843 10.1183/23120541.00074-2016 PMC5395293

[25] E. Schruf , V. Schroeder , H. Q. Le , T. Schönberger , D. Raedel , E. L. Stewart , K. Fundel‐Clemens , T. Bluhmki , S. Weigle , M. Schuler , M. J. Thomas , R. Heilker , M. J. Webster , M. Dass , M. Frick , B. Stierstorfer , K. Quast , J. P. Garnett , FASEB J. 2020, 34, 7825. 32297676 10.1096/fj.201902926R

[26] S. Wang , et al., Biomolecules 2022, 12.

[27] W. Ning , et al., Effect of high glucose supplementation on pulmonary fibrosis involving reactive oxygen species and TGF‐β 2022, 9. 10.3389/fnut.2022.998662 PMC9593073 36304232

[28] C. Y. Ung , A. Onoufriadis , M. Parsons , J. A. McGrath , T. J Shaw , Metabolic perturbations in fibrosis disease 2021, 139, 106073 . 10.1016/j.biocel.2021.106073 34461262

[29] P. He , K. Lim , D. Sun , J. P. Pett , Q. Jeng , K. Polanski , Z. Dong , L. Bolt , L. Richardson , L. Mamanova , M. Dabrowska , A. Wilbrey‐Clark , E. Madissoon , Z. K. Tuong , E. Dann , C. Suo , I. Goh , M. Yoshida , M. Z. Nikolic , S. M. Janes , X. He , R. A. Barker , S. A. Teichmann , J. C. Marioni , K. B. Meyer , E. L. Rawlins , Cell 2022, 185, 4841. 36493756 10.1016/j.cell.2022.11.005 PMC7618435

[30] W.u‐L. Zuo , M. R. Rostami , M. LeBlanc , R. J. Kaner , S. L. O'Beirne , J. G. Mezey , P. L. Leopold , K. Quast , S. Visvanathan , J. S. Fine , M. J. Thomas , R. G. Crystal , PLoS One 2020, 15, e0237529. 32941426 10.1371/journal.pone.0237529 PMC7498242

[31] M. C. Basil , et al., Human distal airways contain a multipotent secretory cell that can regenerate alveoli 2022, 604, 120. 10.1038/s41586-022-04552-0 PMC9297319 35355013

[32] A. C. Habermann , et al., Sci. Adv. 2020, 6, eaba1972. 32832598 10.1126/sciadv.aba1972 PMC7439444

[33] J. E. McDonough , F. Ahangari , Q. Li , S. Jain , S. E. Verleden , J. Herazo‐Maya , M. Vukmirovic , G. DeIuliis , A. Tzouvelekis , N. Tanabe , F. Chu , X. Yan , J. Verschakelen , R. J. Homer , D. V. Manatakis , J. Zhang , J. Ding , K. Maes , L. De Sadeleer , R. Vos , A. Neyrinck , P. V. Benos , Z. Bar‐Joseph , D. Tantin , J. C. Hogg , B. M. Vanaudenaerde , W. A. Wuyts , N. Kaminski , JCI Insight 2019, 4. 10.1172/jci.insight.131597 PMC6948862 31600171

[34] S. Ozcan , S. S. Andrali , J. E. L. Cantrell , Biochim. Biophys. Acta 2010, 1799, 353. 20202486 10.1016/j.bbagrm.2010.02.005 PMC2881704

[35] H. Nie , W. Yi , J Zhejiang Univ Sci B 2019, 20, 437 . 31090269 10.1631/jzus.B1900150 PMC6568225

[36] S. Mizumoto , S. Yamada , Front. Genet. 2021, 12, 717535 . 34539746 10.3389/fgene.2021.717535 PMC8446454

[37] S. E. S. Martin , Z.‐W. Tan , H. M. Itkonen , D. Y. Duveau , J. A. Paulo , J. Janetzko , P. L. Boutz , L. Törk , F. A. Moss , C. J. Thomas , S. P. Gygi , M. B. Lazarus , S. Walker , J. Am. Chem. Soc. 2018, 140, 13542. 30285435 10.1021/jacs.8b07328 PMC6261342

[38] A. V. Leonel , F. Alisson‐Silva , R. C. M. Santos , R. P. Silva‐Aguiar , J. C. Gomes , G. M. C. Longo , B. M. Faria , M. S. Siqueira , M. G. Pereira , A. Vasconcelos‐dos‐Santos , L. B. Chiarini , C. Slawson , C. Caruso‐Neves , L. Romão , L. H. Travassos , K. Carneiro , A. R. Todeschini , W. B. Dias , Cancers 2023, 15, 4740. 37835434 10.3390/cancers15194740 PMC10571858

[39] S. A. Caldwell , et al., Nutrient sensor O‐GlcNAc transferase regulates breast cancer tumorigenesis through targeting of the oncogenic transcription factor FoxM1 2010, 29, 2831. 10.1038/onc.2010.41 20190804

[40] L. Cui , et al., Activation of JUN in fibroblasts promotes pro‐fibrotic programme and modulates protective immunity 2020, 11, 2795. 10.1038/s41467-020-16466-4 PMC7270081 32493933

[41] F.‐J. Ren , X.‐Y. Cai , Y. Yao , G.‐Y. Fang , Front. Cell Infect Microbiol. 2023, 13, 1222265. 37731821 10.3389/fcimb.2023.1222265 PMC10507257

[42] S. Mathas , EMBO J. 2002, 21, 4104. 12145210 10.1093/emboj/cdf389 PMC126136

[43] K. Heinzelmann , Q. Hu , Y. Hu , E. Dobrinskikh , M. Ansari , M. C. Melo‐Narváez , H. M. Ulke , C. Leavitt , C. Mirita , T. Trudeau , M. L. Saal , P. Rice , B. Gao , W. J. Janssen , I. V. Yang , H. B. Schiller , E. K. Vladar , M. Lehmann , M. Königshoff , Eur. Respir. J. 2022, 59, 2102373. 35604813 10.1183/13993003.02373-2021 PMC9203838

[44] E. Wulff‐Fuentes , et al., The human O‐GlcNAcome database and meta‐analysis 2021, 8, 25. 10.1038/s41597-021-00810-4 PMC7820439 33479245

[45] S. P. Moon , A. Javed , E. R. Hard , M. R. Pratt , JACS Au 2022, 2, 74. 35098223 10.1021/jacsau.1c00455 PMC8791055

[46] M. Lam , E. Lamanna , L. Organ , C. Donovan , J. E. Bourke , Front. Pharmacol. 2023, 14, 1162889. 37261291 10.3389/fphar.2023.1162889 PMC10228656

[47] N. J. Lang , J. Gote‐Schniering , D. Porras‐Gonzalez , L. Yang , L. J. De Sadeleer , R. C. Jentzsch , V. A. Shitov , S. Zhou , M. Ansari , A. Agami , C. H. Mayr , B. Hooshiar Kashani , Y. Chen , L. Heumos , J. C. Pestoni , E. S. Molnar , E. Geeraerts , V. Anquetil , L. Saniere , M. Wögrath , M. Gerckens , M. Lehmann , A. Ö. Yildirim , R. Hatz , N. Kneidinger , J. Behr , W. A. Wuyts , M.‐G. Stoleriu , M. D. Luecken , F. J. Theis , et al., Sci. Transl. Med. 2023, 15, eadh0908. 38055803 10.1126/scitranslmed.adh0908

[48] H. N. Alsafadi , C. A. Staab‐Weijnitz , M. Lehmann , M. Lindner , B. Peschel , M. Königshoff , D. E. Wagner , Am J Physiol Lung Cell Mol Physiol 2017, 312, L896. 28314802 10.1152/ajplung.00084.2017

[49] I. Elia , M. C. Haigis , Metabolites and the tumour microenvironment: from cellular mechanisms to systemic metabolism 2021, 3, 21. 10.1038/s42255-020-00317-z PMC8097259 33398194

[50] T. S. Adams , et al., Sci. Adv. 1983, 6, eaba.

[51] P. Yan , J. Liu , Z. Li , J. Wang , Z. Zhu , L. Wang , G. Yu , Int. J. Mol. Sci. 2023, 25, 315. 38203486 10.3390/ijms25010315 PMC10779333

[52] J. Koester , et al., Niche stiffening compromises hair follicle stem cell potential during ageing by reducing bivalent promoter accessibility 2021, 23, 771. 10.1038/s41556-021-00705-x 34239060

[53] M. Segel , et al., Niche stiffness underlies the ageing of central nervous system progenitor cells 2019, 573, 130. 10.1038/s41586-019-1484-9 PMC7025879 31413369

[54] S. Vang , E. S. Helton , Y. Guo , B. Burpee , E. Rose , M. Easter , S. Bollenbecker , M. J. Hirsch , E. L. Matthews , L. I. Jones , P. H. Howze , V. Rajasekaran , R. Denson , P. Cochran , I. K. Attah , H. Olson , G. Clair , G. Melkani , S. Krick , J. W. Barnes , Front. Immunol. 2024, 15, 1387197. 38665916 10.3389/fimmu.2024.1387197 PMC11043510

[55] G. L. Minh , E. M. Esquea , R. G. Young , J. Huang , M. J. Reginato , J. Biol. Chem. 2023, 299, 105344. 37838167 10.1016/j.jbc.2023.105344 PMC10641670

[56] M. Santaguida , K. Schepers , B. King , E. Passegue , Deciphering JunB Function in Regulating Hematopoietic Stem Cell Functions 2007, 110, 777.

[57] K. Singh , E. Camera , L. Krug , A. Basu , R. K. Pandey , S. Munir , M. Wlaschek , S. Kochanek , M. Schorpp‐Kistner , M. Picardo , P. Angel , C. Niemann , P. Maity , K. Scharffetter‐Kochanek , Nat. Commun. 2018, 9, 3425. 30143626 10.1038/s41467-018-05726-z PMC6109099

[58] S. Koizumi , et al., JunB regulates homeostasis and suppressive functions of effector regulatory T cells 2018, 9, 5344. 10.1038/s41467-018-07735-4 PMC6297218 30559442

[59] A. H. Licht , et al., JunB is required for endothelial cell morphogenesis by regulating core‐binding factor β 2006, 175, 981. 10.1083/jcb.200605149 PMC2064707 17158955

[60] B. Pérez‐Benavente , A. Fathinajafabadi , L. de la Fuente , C. Gandía , A. Martínez‐Férriz , J. M. Pardo‐Sánchez , L. Milián , A. Conesa , O. A. Romero , J. Carretero , R. Matthiesen , I. Jariel‐Encontre , M. Piechaczyk , R. Farràs , Genome Biol. 2022, 23, 252. 36494864 10.1186/s13059-022-02800-0 PMC9733061

[61] M. K. Thomsen , et al., Loss of JUNB/AP‐1 promotes invasive prostate cancer 2015, 22, 574. 10.1038/cdd.2014.213 PMC4356344 25526087

[62] Y.‐Y. Liu , et al., O‐GlcNAcylation of MORC2 at threonine 556 by OGT couples TGF‐β signaling to breast cancer progression 2022, 29, 861. 10.1038/s41418-021-00901-0 PMC8991186 34974534

[63] S. E. S. Martin , Z.‐W. Tan , H. M. Itkonen , D. Y. Duveau , J. A. Paulo , J. Janetzko , P. L. Boutz , L. Törk , F. A. Moss , C. J. Thomas , S. P. Gygi , M. B. Lazarus , S. Walker , J. Am. Chem. Soc. 2018, 140, 13542. 30285435 10.1021/jacs.8b07328 PMC6261342

[64] J. A. Gindele , et al., Intermittent exposure to whole cigarette smoke alters the differentiation of primary small airway epithelial cells in the air‐liquid interface culture 2020, 10, 6257. 10.1038/s41598-020-63345-5 PMC7148343 32277131

[65] M. I. Love , W. Huber , S. Anders , Genome Biol. 2014, 15, 550. 25516281 10.1186/s13059-014-0550-8 PMC4302049

[66] E. Y. Chen , C. M. Tan , Y. Kou , Q. Duan , Z. Wang , G. V. Meirelles , N. R. Clark , A. Ma'ayan , BMC Bioinformatics 2013, 14, 128. 23586463 10.1186/1471-2105-14-128 PMC3637064

[67] D. Tang , M. Chen , X. Huang , G. Zhang , L. Zeng , G. Zhang , S. Wu , Y. Wang , PLoS One 18, e0294236. 10.1371/journal.pone.0294236 PMC10635526 37943830

[68] A. Subramanian , P. Tamayo , V. K. Mootha , S. Mukherjee , B. L. Ebert , M. A. Gillette , A. Paulovich , S. L. Pomeroy , T. R. Golub , E. S. Lander , J. P. Mesirov , Proc. National Acad. Sci. 2005, 102, 15545. 10.1073/pnas.0506580102 PMC1239896 16199517

[69] J. D. Buenrostro , P. G. Giresi , L. C. Zaba , H. Y. Chang , W. J. Greenleaf , Nat. Methods 2013, 10, 1213. 24097267 10.1038/nmeth.2688 PMC3959825

[70] H. Patel , nf‐core/atacseq: nf‐core/atacseq v1. 2.2—Iron Ossifrage.

[71] P. A. Ewels , A. Peltzer , S. Fillinger , H. Patel , J. Alneberg , A. Wilm , M. U. Garcia , P. Di Tommaso , S. Nahnsen , Nat. Biotechnol. 2020, 38, 276. 32055031 10.1038/s41587-020-0439-x

[72] F. Ramírez , D. P. Ryan , B. Grüning , V. Bhardwaj , F. Kilpert , A. S. Richter , S. Heyne , F. Dündar , T. Manke , Nucleic Acids Res. 2016, 44, W160. 27079975 10.1093/nar/gkw257 PMC4987876

[73] G. Yu , L.‐G. Wang , Q.‐Y. He , Bioinformatics 2015, 31, 2382. 25765347 10.1093/bioinformatics/btv145

[74] S. Heinz , C. Benner , N. Spann , E. Bertolino , Y. C. Lin , P. Laslo , J. X. Cheng , C. Murre , H. Singh , C. K. Glass , Mol. Cell 2010, 38, 576. 20513432 10.1016/j.molcel.2010.05.004 PMC2898526

[75] J. M. Granja , M. R. Corces , S. E. Pierce , S. T. Bagdatli , H. Choudhry , H. Y. Chang , W. J. Greenleaf , Nat. Genet. 2021, 53, 403. 33633365 10.1038/s41588-021-00790-6 PMC8012210

[76] I. Korsunsky , N. Millard , J. Fan , K. Slowikowski , F. Zhang , K. Wei , Y. Baglaenko , M. Brenner , P.o‐R.u Loh , S. Raychaudhuri , Nat. Methods 2019, 16, 1289. 31740819 10.1038/s41592-019-0619-0 PMC6884693

[77] Y. Zhang , T. Liu , C. A. Meyer , J. Eeckhoute , D. S. Johnson , B. E. Bernstein , C. Nusbaum , R. M. Myers , M. Brown , W. Li , X. S. Liu , Genome Biol. 2008, 9, R137. 18798982 10.1186/gb-2008-9-9-r137 PMC2592715

[78] F. A. Wolf , P. Angerer , F. J. Theis , SCANPY: large‐scale single‐cell gene expression data analysis 2018, 19, 15. 10.1186/s13059-017-1382-0 PMC5802054 29409532

[79] S. L. Wolock , R. Lopez , A. M. S. C. I. C. D. S.‐C. T. D Klein , Cell Syst 2019, 8, 281. 30954476 10.1016/j.cels.2018.11.005 PMC6625319

[80] R. Lopez , J. Regier , M. B. Cole , M. I. Jordan , N. Yosef , Deep generative modeling for single‐cell transcriptomics 2018, 15, 1053. 10.1038/s41592-018-0229-2 PMC6289068 30504886

[81] B. Muzellec , M. Teleńczuk , V. Cabeli , M. Andreux , Bioinformatics 2023, 39, 547. 10.1093/bioinformatics/btad547 PMC10502239 37669147

[82] B. Strobel , et al., Hum. Gene. Ther. Methods 2019, 30, 23. 30693792 10.1089/hgtb.2018.228 PMC6388714

