# Supplementary material for: JUNB O‐GlcNAcylation‐Mediated Promoter Accessibility of Metabolic Genes Modulates Distinct Epithelial Lineage in Pulmonary Fibrosis
Source: Adv Sci (Weinh). 2024 Dec 15;12(5):2406751. doi: 10.1002/advs.202406751 (PMC11791990; doi:10.1002/advs.202406751)
Supplement: Supplementary file 1 — Supporting Information [file ADVS-12-2406751-s004.pdf]

ADVANCED SCIENCE
Open Access

Supporting Information

for *Adv. Sci.*, DOI 10.1002/advs.202406751

JUNB O-GlcNAcylation-Mediated Promoter Accessibility of Metabolic Genes Modulates Distinct Epithelial Lineage in Pulmonary Fibrosis

Marie-Therese Bammert, Meshal Ansari, Leoni Haag, Zuhdi Ahmad, Victoria Schröder, Joseph Birch, Diana Santacruz, Werner Rust, Coralie Viollet, Benjamin Strobel, Alec Dick, Florian Gantner, Holger Schlüter, Fidel Ramirez, Muriel Lizé, Matthew J. Thomas* and Huy Q. Le**

Supporting Information

JUNB O-GlcNAcylation-mediated Promoter Accessibility of Metabolic Genes Modulates Distinct Epithelial Lineage in Pulmonary Fibrosis

Marie-Therese Bammert[1,2], Meshal Ansari[3], Leoni Haag[1], Zuhdi Ahmad[1], Victoria Schröder[1], Joseph Birch[1], Diana Santacruz[3], Werner Rust[3], Coralie Viollet[3], Benjamin Strobel[4], Alec Dick[3], Florian Gantner[2,5], Holger Schlüter[1], Fidel Ramirez[3], Muriel Lizé[1], Matthew J. Thomas[1,6*], Huy Q. Le[1]

Affiliations

[1]Lung Repair & Regeneration Department, Boehringer Ingelheim Pharma GmbH & Co. KG, Biberach, Germany

[2]University of Konstanz, Faculty of Biology, Konstanz, Germany

[3]Global Computational Biology and Digital Science, Boehringer Ingelheim Pharma GmbH & Co. KG, Biberach, Germany

[4]Drug Discovery Sciences, Boehringer Ingelheim Pharma GmbH & Co. KG, Biberach, Germany

[5]C.H. Boehringer Sohn AG and Co. KG, Ingelheim, Germany

[6]University of Bath, Bath, United Kingdom

[*]Correspondence: M.J.T (matthew_james.thomas@boehringer-ingelheim.com)

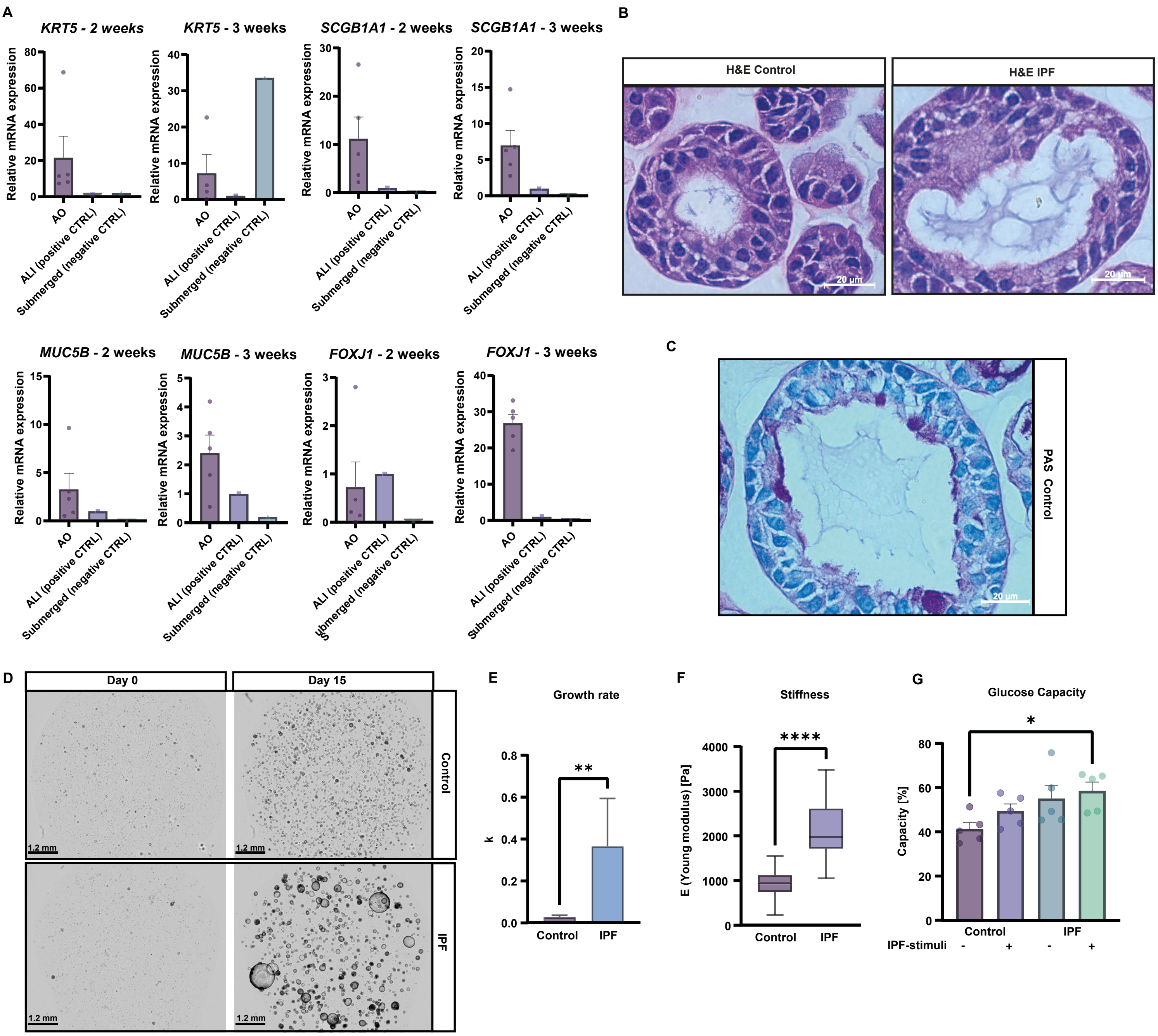
A
KRT5 - 2 weeks
KRT5 - 3 weeks
SCGB1A1 - 2 weeks
SCGB1A1 - 3 weeks
MUC5B - 2 weeks
MUC5B - 3 weeks
FOXJ1 - 2 weeks
FOXJ1 - 3 weeks
Relative mRNA expression
AO
ALI (positive CTRL)
Submerged (negative CTRL)
B
H&E Control
H&E IPF
20 µm
C
PAS Control
D
Day 0
Day 15
Control
IPF
1.2 mm
E
Growth rate
k
Control
IPF
**
F
Stiffness
E (Young modulus) [Pa]
****
G
Glucose Capacity
Capacity [%]
IPF-stimuli
*

Supplemental Fig. S1: Patient-derived AOs recapitulate important fibrotic characteristics.

A, RT-PCR analysis of airway-specific markers *KRT5*, *SCGB1A1*, *MUC5B*, and *FOXJ1* were obtained from n = 5 control AOs (mean + s.e.m) at 2 and 3 weeks of cultivation. Air-liquid interface (ALI) differentiated airway cells serve as positive, while submerged-cultivated airway cells as negative control.

B, Representative H&E staining of IPF and control AOs after 21 days showing lumen and cilia.

C, Representative PAS staining of control AOs after 21 days, confirming glycolytic mucus presence within organoids.

D, Bright-field images of AOs on day 0 and 15 post-seeding, revealing increased size in IPF AOs.

E, Growth rate constant (k) determination from n = 4 IPF and n = 7 control AO donors over 15 days, indicating significantly increased proliferation in IPF AO (mean + s.d, t-test, **$p < 0.01$).

F, Nanoindentation measurement shows increased viscoelasticity (Young modulus) in IPF AOs from n = 5 IPF and n = 5 control donors (boxplot, ****$p < 0.0001$).

G, Increased glucose capacity in IPF AOs (n = 5, mean + s.e.m., *$p < 0.05$, ANOVA/ Tukey's).

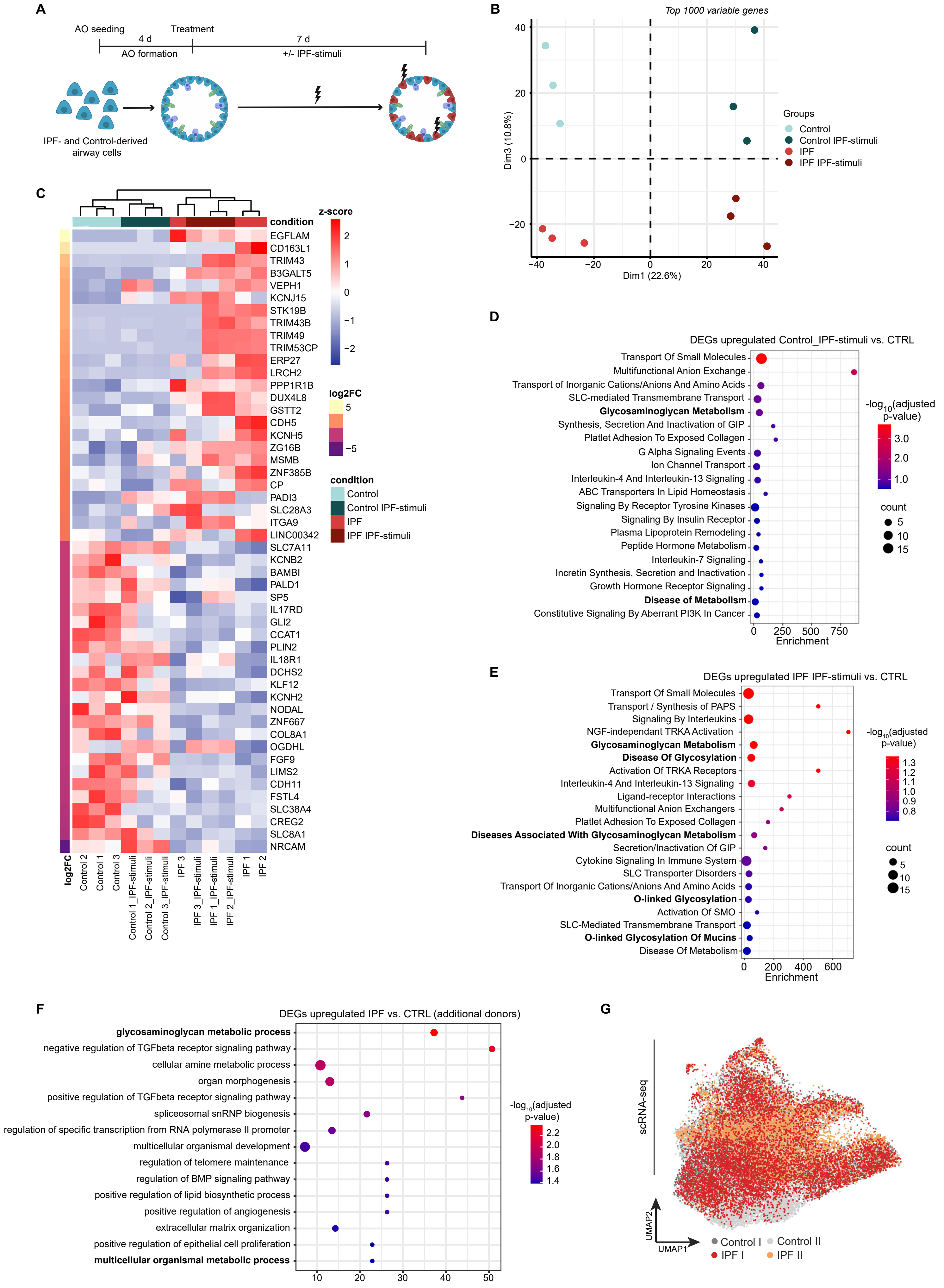
A
AO seeding
Treatment
4 d
7 d
AO formation
+/- IPF-stimuli
IPF- and Control-derived airway cells
B
Top 1000 variable genes
Dim3 (10.8%)
Dim1 (22.6%)
Groups
Control
Control IPF-stimuli
IPF
IPF IPF-stimuli
C
condition
z-score
log2FC
EGFLAM
CD163L1
TRIM43
B3GALT5
VEPH1
KCNJ15
STK19B
TRIM43B
TRIM49
TRIM53CP
ERP27
LRCH2
PPP1R1B
DUX4L8
GSTT2
CDH5
KCNH5
ZG16B
MSMB
ZNF385B
CP
PADI3
SLC28A3
ITGA9
LINC00342
SLC7A11
KCNB2
BAMBI
PALD1
SP5
IL17RD
GLI2
CCAT1
PLIN2
IL18R1
DCHS2
KLF12
KCNH2
NODAL
ZNF667
COL8A1
OGDHL
FGF9
LIMS2
CDH11
FSTL4
SLC38A4
CREG2
SLC8A1
NRCAM
Control 2
Control 1
Control 3
Control 1_IPF-stimuli
Control 2_IPF-stimuli
Control 3_IPF-stimuli
IPF 3
IPF 3_IPF-stimuli
IPF 1_IPF-stimuli
IPF 2_IPF-stimuli
IPF 1
IPF 2
condition
Control
Control IPF-stimuli
IPF
IPF IPF-stimuli
D
DEGs upregulated Control_IPF-stimuli vs. CTRL
Transport Of Small Molecules
Multifunctional Anion Exchange
Transport of Inorganic Cations/Anions And Amino Acids
SLC-mediated Transmembrane Transport
Glycosaminoglycan Metabolism
Synthesis, Secretion And Inactivation of GIP
Platlet Adhesion To Exposed Collagen
G Alpha Signaling Events
Ion Channel Transport
Interleukin-4 And Interleukin-13 Signaling
ABC Transporters In Lipid Homeostasis
Signaling By Receptor Tyrosine Kinases
Signaling By Insulin Receptor
Plasma Lipoprotein Remodeling
Peptide Hormone Metabolism
Interleukin-7 Signaling
Incretin Synthesis, Secretion and Inactivation
Growth Hormone Receptor Signaling
Disease of Metabolism
Constitutive Signaling By Aberrant PI3K In Cancer
Enrichment
-log10(adjusted p-value)
count
E
DEGs upregulated IPF IPF-stimuli vs. CTRL
Transport Of Small Molecules
Transport / Synthesis of PAPS
Signaling By Interleukins
NGF-independant TRKA Activation
Glycosaminoglycan Metabolism
Disease Of Glycosylation
Activation Of TRKA Receptors
Interleukin-4 And Interleukin-13 Signaling
Ligand-receptor Interactions
Multifunctional Anion Exchangers
Platlet Adhesion To Exposed Collagen
Diseases Associated With Glycosaminoglycan Metabolism
Secretion/Inactivation Of GIP
Cytokine Signaling In Immune System
SLC Transporter Disorders
Transport Of Inorganic Cations/Anions And Amino Acids
O-linked Glycosylation
Activation Of SMO
SLC-Mediated Transmembrane Transport
O-linked Glycosylation Of Mucins
Disease Of Metabolism
Enrichment
-log10(adjusted p-value)
count
F
DEGs upregulated IPF vs. CTRL (additional donors)
glycosaminoglycan metabolic process
negative regulation of TGFbeta receptor signaling pathway
cellular amine metabolic process
organ morphogenesis
positive regulation of TGFbeta receptor signaling pathway
spliceosomal snRNP biogenesis
regulation of specific transcription from RNA polymerase II promoter
multicellular organismal development
regulation of telomere maintenance
regulation of BMP signaling pathway
positive regulation of lipid biosynthetic process
positive regulation of angiogenesis
extracellular matrix organization
positive regulation of epithelial cell proliferation
multicellular organismal metabolic process
-log10(adjusted p-value)
G
scRNA-seq
UMAP2
UMAP1
Control I
Control II
IPF I
IPF II

Supplemental Fig. S2: Aberrant OGlcNAcylation is involved in IPF.

A, Schematic representation of the experimental procedure. Singularised AOs were seeded and cultivated for 4 days to induce organoid formation and grown for another 7 days. Additionally, they could be exposed to IPF stimuli for 7 days to increase the fibrotic phenotype. After a total of 11 days, AOs were collected and processed for multiome bulk RNA-/ATAC-seq or scATAC-seq.

B, PCA of RNA-seq data with the top 1000 variable genes (q-value < 0.05) according to coefficient of variation shows distinct clustering of IPF and control AOs.

C, Hierarchical clustering of top 50 DEGs from RNA-seq comparing control and IPF AOs including treatment with IPF-stimuli (q-value < 0.05).

D, Reactome analysis of IPF-stimulated control AOs.

E, Reactome analysis of IPF-stimulated IPF AOs.

F, Reactome analysis of expanded data set to n = 5 new control and n = 5 new IPF AO donors shows again enrichment of glycosaminoglycan metabolic process further validating previously generated data set of n = 3 ($p < 0.05$, Log2FC > 0).

G, Reduced-dimensionality (UMAP) visualization of n = 2 control and n = 2 IPF single cells from AOs in transcriptome space.

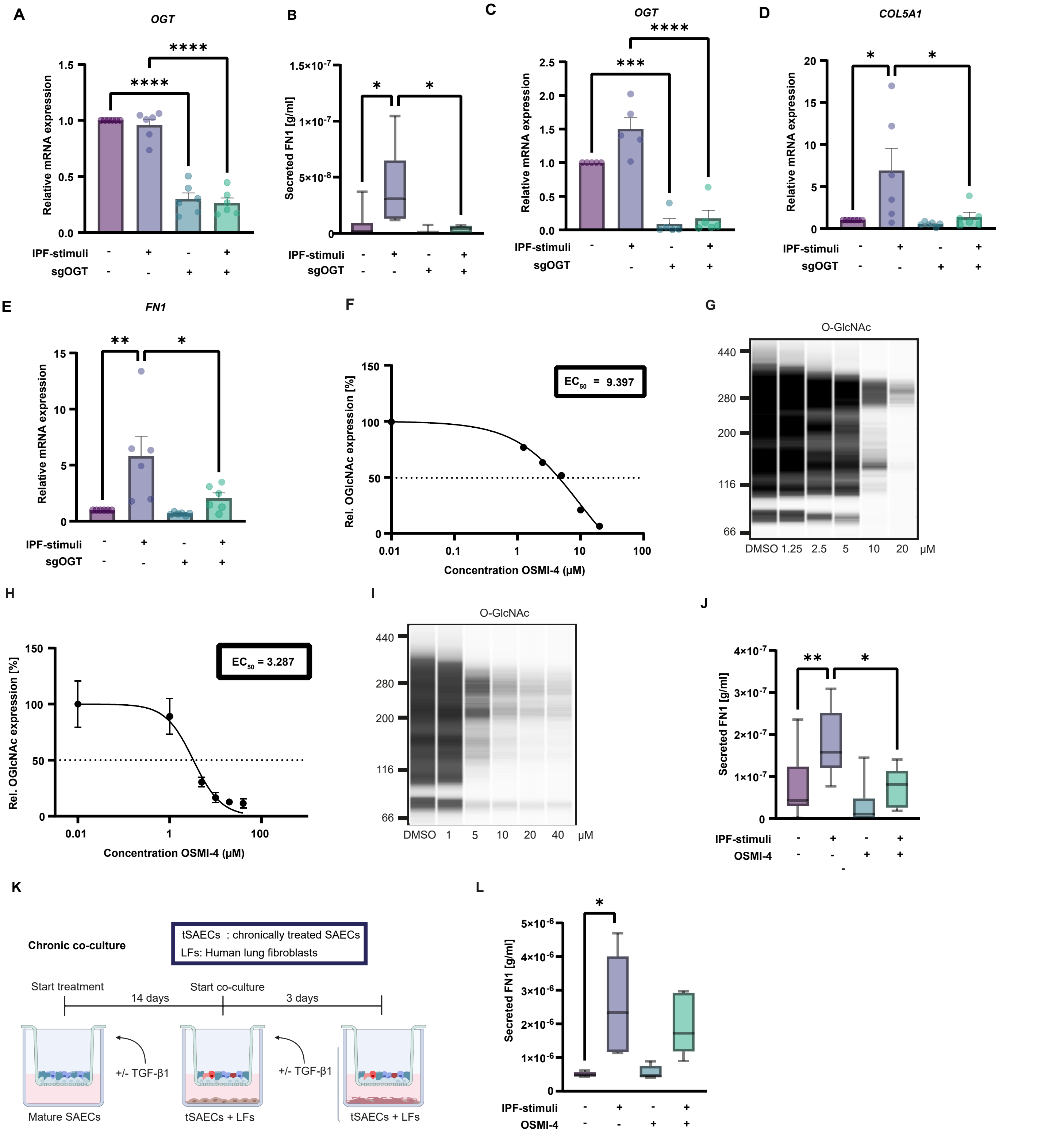
A
OGT
Relative mRNA expression
IPF-stimuli
sgOGT
B
Secreted FN1 [g/ml]
C
OGT
D
COL5A1
E
FN1
F
EC50 = 9.397
Rel. OGlcNAc expression [%]
Concentration OSMI-4 (µM)
G
O-GlcNAc
DMSO 1.25 2.5 5 10 20 µM
H
EC50 = 3.287
I
O-GlcNAc
DMSO 1 5 10 20 40 µM
J
OSMI-4
K
Chronic co-culture
tSAECs : chronically treated SAECs
LFs: Human lung fibroblasts
Start treatment
14 days
Start co-culture
3 days
+/- TGF-β1
Mature SAECs
tSAECs + LFs
L

Supplemental Fig. S3: O-GlcNAcylation plays an important regulatory role in IPF.

A, RT-PCR shows that OGT deletion in airway epithelial cells leads to decrease in *OGT* expression after IPF stimulation (n = 6, mean + s.e.m, ****$p < 0.0001$, ANOVA/ Tukey's).

B, ELISA analysis represents a decrease in pro-fibrotic protein secretion of FN1 in OGT-deleted airway epithelial cells stimulated with IPF-stimuli. (n = 8, boxplot, *p < 0.05, ANOVA/ Friedman).

C, RT-PCR shows that OGT deletion in lung fibroblasts treated with fibrotic stimuli leads to decrease in OGT expression (n = 6, mean + s.e.m, ***p < 0.001, ****p < 0.0001, ANOVA/ Tukey's).

D, E, RT-PCR reveals attenuated expression of profibrotic genes *COL5A1* (**D**) and *FN1* (**E**) upon OGT deletion in fibroblasts after fibrotic induction with IPF-stimuli (n = 6, mean + s.e.m, *$p < 0.05$, **$p < 0.01$, ANOVA/ Tukey's).

F, G, Determination of EC_{50} values for OSMI-4 in AOs based on relative O-GlcNAc expression from western blot analysis (**G**) and quantification (**F**) of AO protein lysates treated within a range of 1.25-20 µM OSMI-4 for 7 days. DMSO was used as negative control.

H, **I**, Determination of EC_{50} values for OSMI-4 in airway epithelial cells based on relative O-GlcNAc expression from western blot analysis (**I**) and quantification (**H**) of AO protein lysates treated within a range of 1.0-40 µM OSMI-4 for 72 h. DMSO was used as negative control.

J, ELISA analysis shows a decrease in pro-fibrotic protein secretion of FN1 in OGT-inhibited airway epithelial cells stimulated with IPF-inducing stimuli. (n = 5, boxplot, *p < 0.05, **p < 0.01, ANOVA/Tukey's).

K, Schematic overview of epithelial-mesenchymal-coculture set up and treatment strategy. Airway epithelial cells were grown for 10 days in air-liquid-interface, before being exposed to IPF-stimuli for another 14 days to induce chronic injury. Afterwards, injured airway epithelial cells (tSAECs) and lung fibroblasts were cocultured for another 72 h.

L, ELISA analysis reveals a decrease in pro-fibrotic protein secretion of FN1 in OGT-inhibited epithelial-fibroblast coculture secretome stimulated with IPF-inducing stimuli. (n = 5, boxplot, *$p < 0.05$, ANOVA/Holm-Šídák's).

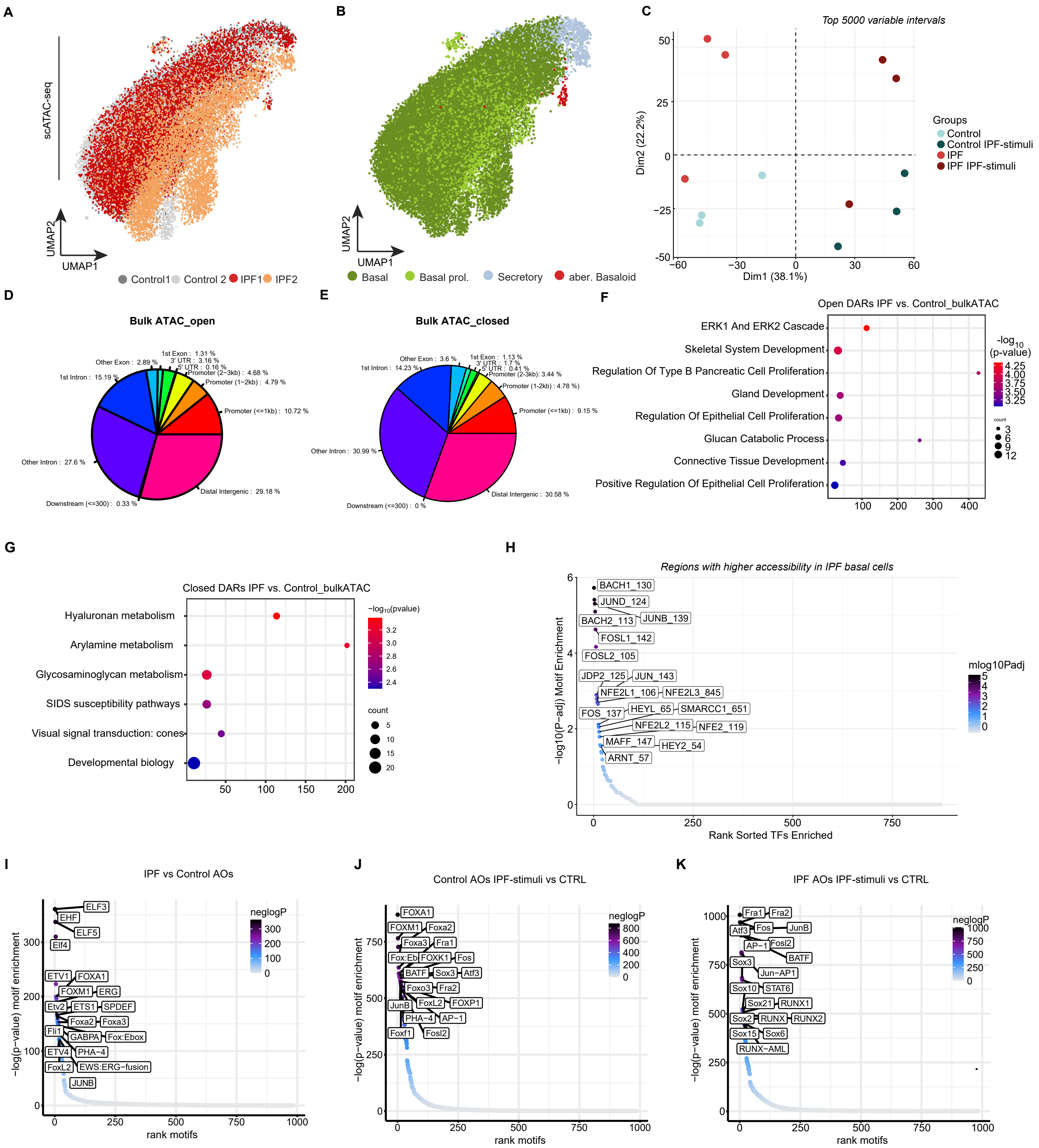
A
scATAC-seq
UMAP2
UMAP1
Control1
Control 2
IPF1
IPF2
B
UMAP2
UMAP1
Basal
Basal prol.
Secretory
aber. Basaloid
C
Top 5000 variable intervals
Dim2 (22.2%)
Dim1 (38.1%)
Groups
Control
Control IPF-stimuli
IPF
IPF IPF-stimuli
D
Bulk ATAC_open
1st Exon : 1.31 %
3' UTR : 3.16 %
5' UTR : 0.16 %
Other Exon : 2.89 %
Promoter (2−3kb) : 4.68 %
Promoter (1−2kb) : 4.79 %
1st Intron : 15.19 %
Promoter (<=1kb) : 10.72 %
Other Intron : 27.6 %
Distal Intergenic : 29.18 %
Downstream (<=300) : 0.33 %
E
Bulk ATAC_closed
Other Exon : 3.6 %
1st Exon : 1.13 %
3' UTR : 1.7 %
5' UTR : 0.41 %
1st Intron : 14.23 %
Promoter (2-3kb): 3.44 %
Promoter (1-2kb) : 4.78 %)
Promoter (<=1kb) : 9.15 %
Other Intron : 30.99 %
Distal Intergenic : 30.58 %
Downstream (<=300) : 0 %
F
Open DARs IPF vs. Control_bulkATAC
ERK1 And ERK2 Cascade
Skeletal System Development
Regulation Of Type B Pancreatic Cell Proliferation
Gland Development
Regulation Of Epithelial Cell Proliferation
Glucan Catabolic Process
Connective Tissue Development
Positive Regulation Of Epithelial Cell Proliferation
-log10 (p-value)
count
G
Closed DARs IPF vs. Control_bulkATAC
Hyaluronan metabolism
Arylamine metabolism
Glycosaminoglycan metabolism
SIDS susceptibility pathways
Visual signal transduction: cones
Developmental biology
−log10(pvalue)
count
H
Regions with higher accessibility in IPF basal cells
BACH1_130
JUND_124
BACH2_113
JUNB_139
FOSL1_142
FOSL2_105
JDP2_125
JUN_143
NFE2L1_106
NFE2L3_845
FOS_137
HEYL_65
SMARCC1_651
NFE2L2_115
NFE2_119
MAFF_147
HEY2_54
ARNT_57
−log10(P−adj) Motif Enrichment
Rank Sorted TFs Enriched
mlog10Padj
I
IPF vs Control AOs
ELF3
EHF
ELF5
Elf4
ETV1
FOXA1
FOXM1
ERG
Etv2
ETS1
SPDEF
Foxa2
Foxa3
Fli1
GABPA
Fox:Ebox
ETV4
PHA−4
FoxL2
EWS:ERG−fusion
JUNB
−log(p−value) motif enrichment
rank motifs
neglogP
J
Control AOs IPF-stimuli vs CTRL
FOXA1
FOXM1
Foxa2
Foxa3
Fra1
Fox:Eb
FOXK1
Fos
BATF
Sox3
Atf3
Foxo3
Fra2
JunB
FoxL2
FOXP1
PHA−4
AP−1
Foxf1
Fosl2
−log(p−value) motif enrichment
rank motifs
neglogP
K
IPF AOs IPF-stimuli vs CTRL
Fra1
Fra2
Atf3
Fos
JunB
AP−1
Fosl2
BATF
Sox3
Jun−AP1
Sox10
STAT6
Sox21
RUNX1
Sox2
RUNX
RUNX2
Sox15
Sox6
RUNX−AML
−log(p−value) motif enrichment
rank motifs
neglogP

Supplemental Fig. S4: JUNB TF motif enrichment in IPF basal cells promotes fibrotic cell fate.

A, **B**, UMAP representation of single-cell chromatin accessibility (scATAC-seq) clustering in IPF and control AOs (**A**) and IPF cell types (**B**) (basal, proliferating basal, secretory and aberrant basaloid cells).

C, PCA of top 5000 variable regions (bulk ATAC-seq) (*q*-value < 0.05) according to coefficient of variation shows distinct clustering of IPF and control-derived AOs with and without IPF-RC treatment.

D, **E**, Genomic annotations of IPF AOs (bulk ATAC-seq) open (**D**) and closed (**E**) differential accessible regions (DARs, *q*-value < 0.05) reveals enriched open chromatin in IPF promotor regions close to the transcription start site (TSS).

F, GO enrichment analysis of protein coding, open DARs (*q*-value < 0.05) in IPF AOs (bulk ATAC-seq) shows involvement of developmental and proliferative pathways.

G, Bioplanet analysis of closed DARs (*q*-value < 0.05) in IPF AOs (bulk ATAC-seq) shows enrichment of metabolic pathways.

H, Transcription factor (TF) motif enrichment analysis shows upregulation of different activator protein 1 (AP-1) motifs in IPF basal cells (scATAC-seq).

I - K, TF motif enrichment analysis (bulk ATAC-seq) in IPF vs. Control AOs (**I**), Control AOs treated with IPF-stimuli vs. CTRL (**J**), and IPF AOs treated with IPF-stimuli vs. CTRL (**K**) reveals increased motif enrichment for AP-1 family members in IPF conditions.

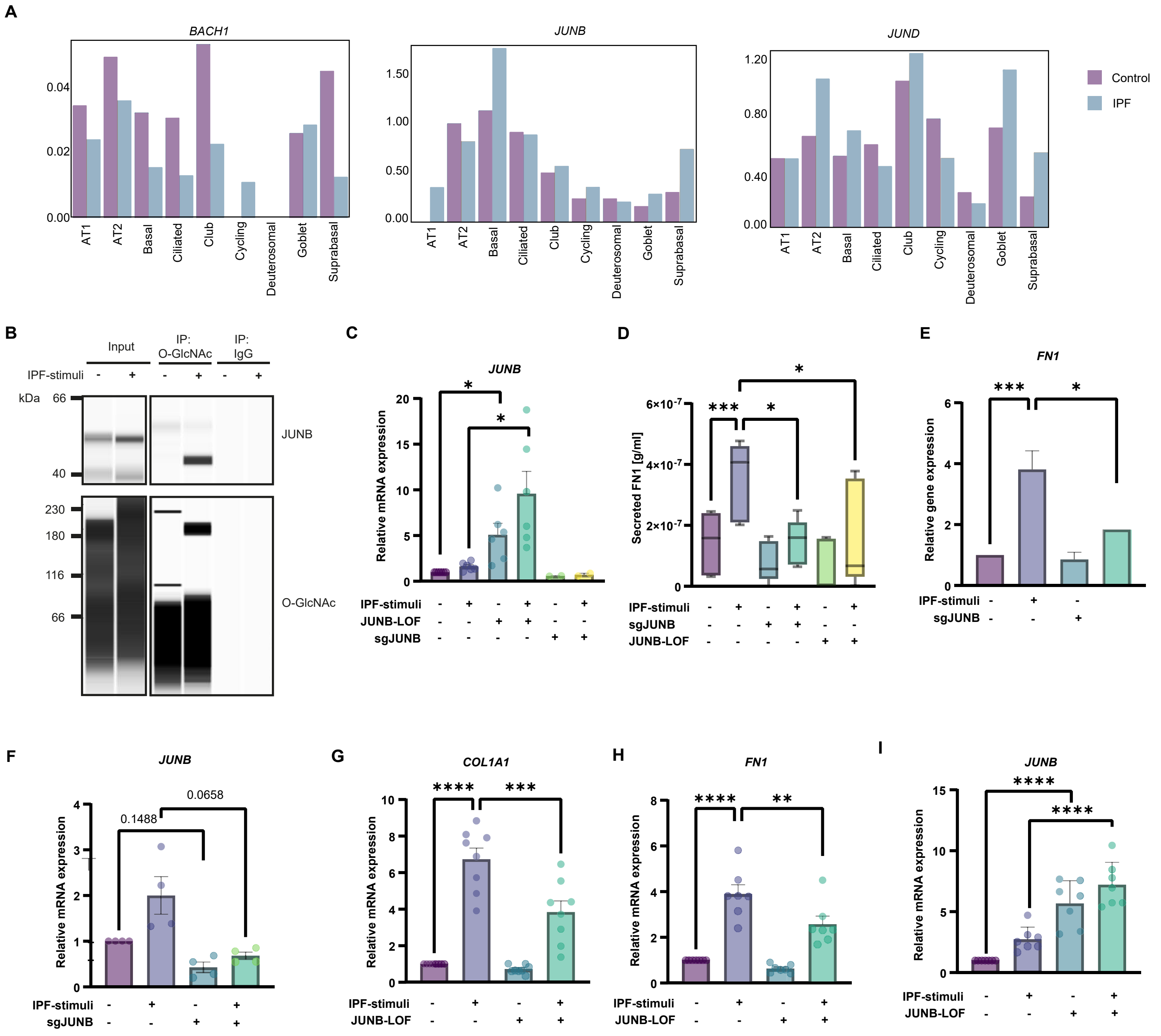
A
BACH1
JUNB
JUND
Control
IPF
AT1
AT2
Basal
Ciliated
Club
Cycling
Deuterosomal
Goblet
Suprabasal
B
Input
IP: O-GlcNAc
IP: IgG
IPF-stimuli
kDa
JUNB
O-GlcNAc
C
JUNB
Relative mRNA expression
IPF-stimuli
JUNB-LOF
sgJUNB
D
Secreted FN1 [g/ml]
IPF-stimuli
sgJUNB
JUNB-LOF
E
FN1
Relative gene expression
IPF-stimuli
sgJUNB
F
JUNB
0.1488
0.0658
Relative mRNA expression
IPF-stimuli
sgJUNB
G
COL1A1
Relative mRNA expression
IPF-stimuli
JUNB-LOF
H
FN1
Relative mRNA expression
IPF-stimuli
JUNB-LOF
I
JUNB
Relative mRNA expression
IPF-stimuli
JUNB-LOF

Supplemental Fig. S5: O-GlcNAcylation of JUNB is involved in the pro-fibrotic response.

A, Expression levels of the top TFs in different epithelial cells. Notably, *JUNB* shows highest differential expression in IPF basal cells.

B, Representative simple western analysis of O-GlcNAc immunoprecipitated fraction showing increase of O-GlcNAc mark on JUNB in injured n = 3 lung fibroblast donors. IgG antibody was used as negative control.

C, RT-PCR reveals increased expression levels of *JUNB* after JUNB-LOF induction and decreased expression levels after JUNB deletion (n = 6, mean + s.e.m, *p < 0.05).

D, ELISA analysis shows a decrease in the secretion of pro-fibrotic marker FN1 in JUNB-LOF and JUNB-deleted airway epithelial cells treated with IPF-inducing stimuli. (n = 5, boxplot, *p < 0.05, ***p < 0.001 ANOVA/Holm-Šídák's).

E, RT-PCR reveals attenuated expression of profibrotic gene FN1 upon JUNB deletion in lung fibroblasts after IPF-stimuli treatment (n = 6, mean + s.e.m, p* < 0.05, p*** < 0.001, ANOVA/Tukey’s).

F, RT-PCR shows decreased *JUNB* expression upon JUNB deletion in lung fibroblasts after fibrotic treatment with IPF-stimuli (n = 5, mean + s.e.m, ANOVA/Tukey’s).

G, H, RT-PCR shows reduced fibrotic gene expression upon JUNB-LOF transfection and fibrotic treatment with IPF-stimuli representatively shown for *COL1A1* (**G**) and *FN1* (**H**) in lung fibroblasts (n = 7, mean + s.e.m, **p < 0.01, ***p < 0.001, ****p < 0.0001, ANOVA/Tukey’s).

I, RT-PCR reveals increased *JUNB* levels upon induction of JUNB-LOF in lung fibroblasts (n = 7, mean + s.e.m, ****p < 0.0001, ANOVA/ Tukey’s).

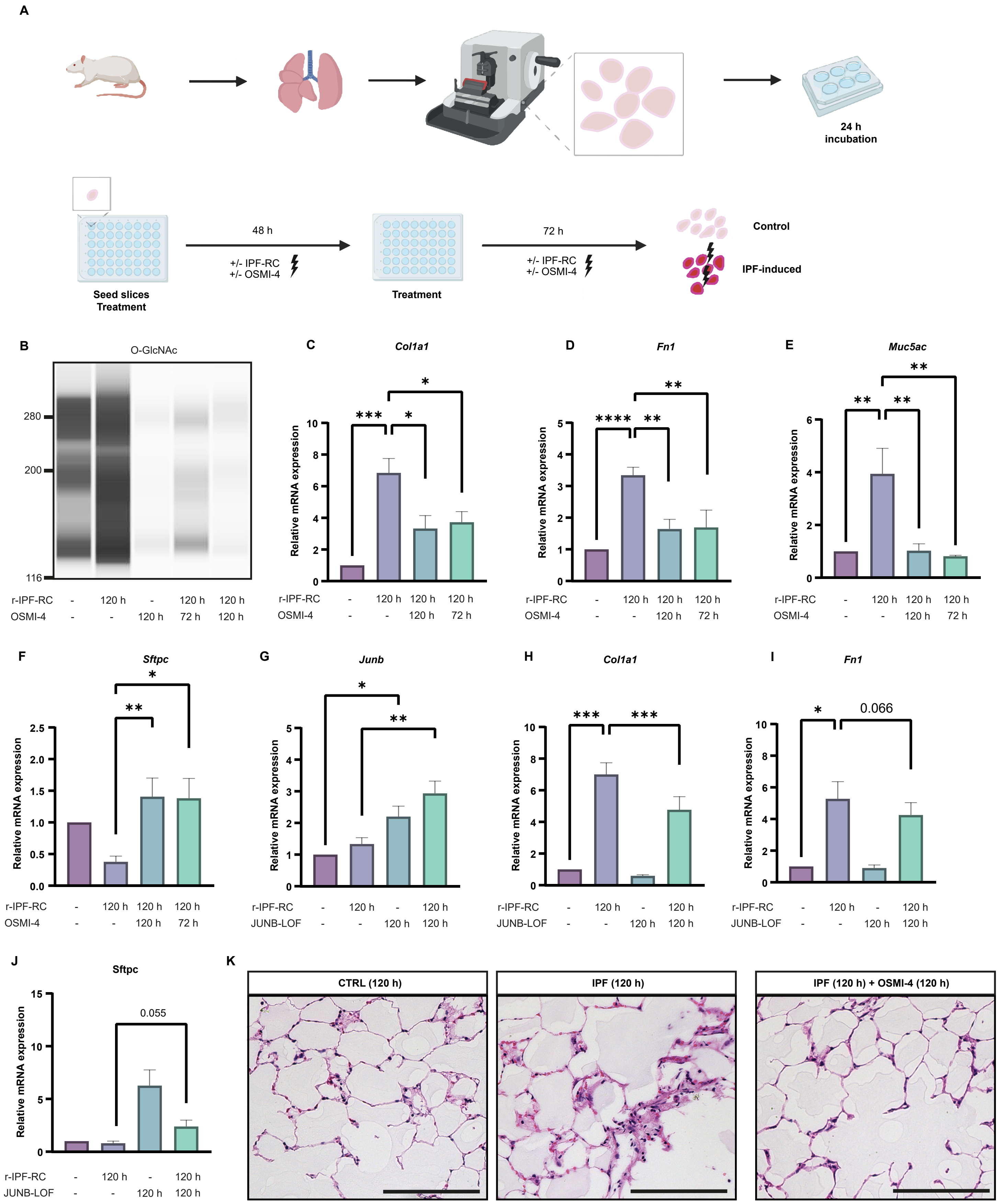
A
24 h
incubation
Seed slices
Treatment
48 h
+/- IPF-RC
+/- OSMI-4
Treatment
72 h
+/- IPF-RC
+/- OSMI-4
Control
IPF-induced
B
O-GlcNAc
280
200
116
r-IPF-RC - 120 h - 120 h 120 h
OSMI-4 - - 120 h 72 h 120 h
C
Col1a1
Relative mRNA expression
r-IPF-RC - 120 h 120 h 120 h
OSMI-4 - - 120 h 72 h
D
Fn1
Relative mRNA expression
r-IPF-RC - 120 h 120 h 120 h
OSMI-4 - - 120 h 72 h
E
Muc5ac
Relative mRNA expression
r-IPF-RC - 120 h 120 h 120 h
OSMI-4 - - 120 h 72 h
F
Sftpc
Relative mRNA expression
r-IPF-RC - 120 h 120 h 120 h
OSMI-4 - - 120 h 72 h
G
Junb
Relative mRNA expression
r-IPF-RC - 120 h - 120 h
JUNB-LOF - - 120 h 120 h
H
Col1a1
Relative mRNA expression
r-IPF-RC - 120 h - 120 h
JUNB-LOF - - 120 h 120 h
I
Fn1
0.066
Relative mRNA expression
r-IPF-RC - 120 h - 120 h
JUNB-LOF - - 120 h 120 h
J
Sftpc
0.055
Relative mRNA expression
r-IPF-RC - 120 h - 120 h
JUNB-LOF - - 120 h 120 h
K
CTRL (120 h)
IPF (120 h)
IPF (120 h) + OSMI-4 (120 h)

Supplemental Fig. S6: OSMI-4 inhibits O-GlcNAc via blocking OGT in rPCLS.

A, Schematic overview of generation and treatment of rPCLS. Healthy rats were euthanized, lungs were filled with agarose and lung tissue dissected. Slices were cut with a microtome to a thickness of 200-350 µm. Afterwards, slices were incubated for 24 h prior to seeding and treatment with +/- r-IPF-RC and +/- OSMI-4 for 48 h followed by additional treatment for another 72 h (total treatment time: 120 h).

B, Representative simple western analysis of O-GlcNAc fraction in rPCLS treated with +/- r-IPF-RC and +/- OSMI-4 showed increase of O-GlcNAc mark after stimulation with r-IPF-RC for 120 h and decreased O-GlcNAc after addition of OSMI-4 for 120 h or 72 h.

C-F, RT-PCR analysis of *Col1a1* (**C**), *Fn1* (**D**), *Muc5ac* (**E**), and *Sftpc* (**F**) in rPCLS with r-IPF-RC and with OSMI-4 for 120 h showed reduction in fibrotic response and induction of regeneration compared to control (n = 5 rats, 2 slices per rat, mean + s.e.m, $*p < 0.05$, $**p < 0.01$, $***p < 0.001$, $****p < 0.0001$, ANOVA/Tukey's).

G, RT-PCR analysis of *Junb* in rPCLS transduced with JUNB-LOF for a total of 120 h showed increase of expression (n = 3 rats, 2 slices per rat, mean + s.e.m. $*p < 0.05$, $**p < 0.01$, ANOVA/Holm-Šídák's)

H, RT-PCR analysis of *Col1a1* in rPCLS transduced with JUNB-LOF for a total of 120 h and co-stimulation with rIPF-RC showed decrease of expression (n = 3 rats, 2 slices per rat, mean + s.e.m. $***p < 0.001$, ANOVA/Bonferroni)

I, RT-PCR analysis of *Fn1* in rPCLS transduced with JUNB-LOF for a total of 120 h and co-stimulation with rIPF-RC showed decrease of expression (n = 3 rats, 2 slices per rat, mean + s.e.m. $*q < 0.05$, ANOVA/Benjamini)

J, RT-PCR analysis of *Fn1* in rPCLS transduced with JUNB-LOF for a total of 120 h and co-stimulation with rIPF-RC showed decrease of expression (n = 3 rats, 2 slices per rat, mean + s.e.m., ANOVA/FDR)

K, Representative H&E stainings of rPCLS slices after respective t reatment for 120 h shows reduction of fibrotic lung architecture upon OSMI-4 treatment for 120 h (scale bar 150 μm).
